# Human Colon Cancer–Derived *Clostridioides difficile* Strains Drive Colonic Tumorigenesis in Mice

**DOI:** 10.1158/2159-8290.CD-21-1273

**Published:** 2022-06-09

**Authors:** Julia L. Drewes, Jie Chen, Nicholas O. Markham, Reece J. Knippel, Jada C. Domingue, Ada J. Tam, June L. Chan, Lana Kim, Madison McMann, Courtney Stevens, Christine M. Dejea, Sarah Tomkovich, John Michel, James R. White, Fuad Mohammad, Victoria L. Campodónico, Cody N. Heiser, Xinqun Wu, Shaoguang Wu, Hua Ding, Patricia Simner, Karen Carroll, Martha J. Shrubsole, Robert A. Anders, Seth T. Walk, Christian Jobin, Fengyi Wan, Robert J. Coffey, Franck Housseau, Ken S. Lau, Cynthia L. Sears

**Affiliations:** 1 Division of Infectious Diseases, Department of Medicine, Johns Hopkins University School of Medicine, Baltimore, Maryland.; 2 Department of Molecular Microbiology and Immunology, Bloomberg School of Public Health, Baltimore, Maryland.; 3 Division of Gastroenterology, Department of Medicine, Vanderbilt University Medical Center, Nashville, Tennessee.; 4 Epithelial Biology Center, Vanderbilt University School of Medicine, Nashville, Tennessee.; 5 Sidney Kimmel Comprehensive Cancer Center, Johns Hopkins University School of Medicine, Baltimore, Maryland.; 6 Department of Oncology, Johns Hopkins University School of Medicine, Baltimore, Maryland.; 7 Department of Medicine, University of Florida, Gainesville, Florida.; 8 Resphera Biosciences, Baltimore, Maryland.; 9 Department of Cell and Developmental Biology and Program in Chemical and Physical Biology, Vanderbilt University School of Medicine, Nashville, Tennessee.; 10 Department of Pathology, Johns Hopkins University School of Medicine, Baltimore, Maryland.; 11 Vanderbilt Ingram Cancer Center, Nashville, Tennessee.; 12 Division of Epidemiology, Department of Medicine, Vanderbilt University Medical Center, Nashville, Tennessee.; 13 Department of Microbiology and Cell Biology, Montana State University, Bozeman, Montana.; 14 Department of Anatomy and Cell Biology, University of Florida, Gainesville, Florida.; 15 Department of Infectious Diseases and Immunology, University of Florida, Gainesville, Florida.; 16 Department of Biochemistry and Molecular Biology, Bloomberg School of Public Health, Johns Hopkins Medical Institutions, Baltimore, Maryland.

## Abstract

Defining the complex role of the microbiome in colorectal cancer and the discovery of novel, protumorigenic microbes are areas of active investigation. In the present study, culturing and reassociation experiments revealed that toxigenic strains of *Clostridioides difficile* drove the tumorigenic phenotype of a subset of colorectal cancer patient–derived mucosal slurries in germ-free *Apc^Min/+^* mice. Tumorigenesis was dependent on the *C. difficile* toxin TcdB and was associated with induction of Wnt signaling, reactive oxygen species, and protumorigenic mucosal immune responses marked by the infiltration of activated myeloid cells and IL17-producing lymphoid and innate lymphoid cell subsets. These findings suggest that chronic colonization with toxigenic *C. difficile* is a potential driver of colorectal cancer in patients.

**Significance:**

Colorectal cancer is a leading cause of cancer and cancer-related deaths worldwide, with a multifactorial etiology that likely includes procarcinogenic bacteria. Using human colon cancer specimens, culturing, and murine models, we demonstrate that chronic infection with the enteric pathogen *C. difficile* is a previously unrecognized contributor to colonic tumorigenesis.

*See related commentary by Jain and Dudeja, p. 1838.*

*This article is highlighted in the In This Issue feature, p. 1825*

## Teaser

The patient-derived *Clostridioides difficile* bacterial strain was found to drive colon tumorigenesis through its toxin TcdB, which induced Wnt signaling and a protumorigenic mucosal immune response.

## INTRODUCTION

The gut microbiome is implicated in the initiation and progression of colorectal cancer. Colorectal cancer is associated with marked fecal and mucosal dysbiosis (1–3), and colonic tumors can be induced in susceptible mouse models by gavage with fecal or colonic mucosal slurries from patients with colorectal cancer and even some healthy individuals (4–6). A limited number of bacteria including enterotoxigenic *Bacteroides fragilis* (ETBF), colibactin-producing *Escherichia coli* (pks^+^Ec), and oral fusobacteria strains have been associated with colorectal cancer in cross-sectional clinical cohorts and drive tumorigenesis in animal models (7–9). However, other protumorigenic organisms may exist.

We previously established that human colorectal cancers are enriched for mucus-invasive microbial biofilms (BF) that interact directly with the colonic epithelium and are associated with procarcinogenic changes, including elevations in Ki-67, pSTAT3, and the polyamine N_1_,N_12_-diacetylspermine (1, 10, 11). Mixed colonic mucosal slurries derived from five patients with BF^+^ colorectal cancer or BF^+^ healthy patients induced robust tumorigenesis in the distal colons of germ-free (GF) *Apc^Min/+^* and *Apc^Min/+^;Il10^−/−^* mice, whereas BF^−^ mucosal slurries from individuals undergoing colonoscopy were not tumorigenic (5). However, whether individual bacteria or the entire BF community is required for tumor induction remains unclear.

Herein, we tested the tumorigenic potential of individual human colorectal cancer mucosal slurries and identified, through microbiological and sequencing methods, a procarcinogenic bacterial consortium in which carcinogenesis was dependent, unexpectedly, on *Clostridioides difficile* and its toxin, TcdB. Importantly, toxigenic (TcdA^+^TcdB^+^) *C. difficile* strains were isolated from both a BF^+^ and BF^−^ human colorectal cancer sample. The presence of *C. difficile* markedly modified colonic epithelial progenitor cells, a potential initiating cell of colorectal cancer, and mucosal immune responses. The complex colonic epithelial changes identified *in vivo* are consistent with the potential for *C. difficile* to promote colonic carcinogenesis.

## RESULTS

### Individual Colorectal Cancer Patient Mucosal Slurries Display Variable Tumorigenic Potential

**Figure 1.**
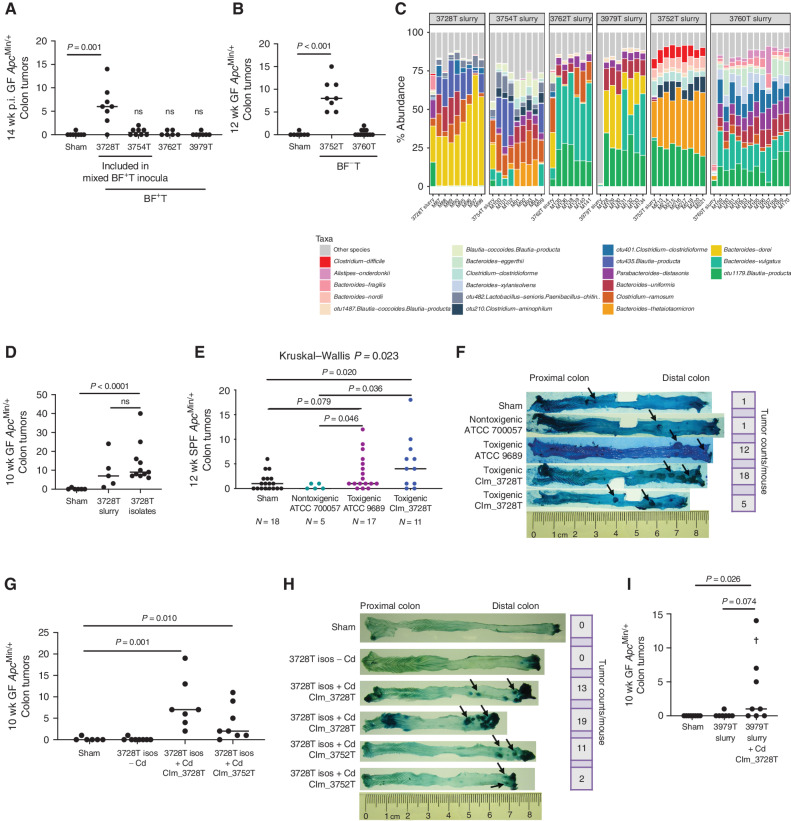
*C. difficile* strains isolated from human colon cancer mucosa drive colonic tumor formation in GF *Apc^Min/+^* mice. **A** and **B,** Variation in colonic tumorigenic potential in GF *Apc^Min/+^* mice at 12 to 14 weeks post-inoculation (p.i.) by individual BF-positive tumor (BF^+^T; **A**) or BF-negative tumor (BF^−^T; **B**) colorectal cancer mucosal samples from six patients. *n* = 6–8 mice per group, from two independent experiments. **C,** 16S rRNA amplicon sequencing relative abundances of the top 20 species in the six patient tumor slurry inocula and 12 to 14 weeks p.i. stools from mice gavaged with the tumor slurries. **D,** A consortium of 30 bacterial isolates derived from GF mice gavaged with the 3728T slurry recapitulated colonic tumorigenesis of the original 3728T slurry. *n* = 5–11 mice per group. **E** and **F,** Colonic tumor counts (**E**) and representative methylene blue staining (**F**) of colonic tumors (arrows) at 12 weeks p.i. in a vancomycin/gentamicin model (see Methods) of chronic colonization with nontoxigenic (TcdA^−^TcdB^−^) or toxigenic (TcdA^+^TcdB^+^) *C. difficile* strains. *n* = 5–18 mice per group, from two independent experiments. **G** and **H,** Colonic tumor counts (**G**) and representative methylene blue staining (**H**) of colonic tumors (arrows) from GF *Apc^Min/+^* mice gavaged with the 3728T isolates with or without the CIm_3728T or CIm_3752T *C. difficile* strains. *n* = 6–8 mice per group. **I,** Colonic tumors induced in GF *Apc^Min/+^* mice gavaged with either the 3979T slurry or the 3979T slurry with *C. difficile* strain CIm_3728T at 10 weeks p.i. The cross represents a mouse death. *n* = 7–8 mice per group. Statistical significance was calculated via Kruskal–Wallis followed by Mann–Whitney tests. Mann–Whitney *P* values are shown. ns, not significant.

We first tested individual patient-derived colorectal cancer slurries in *Apc^Min/+^* mice to examine their tumorigenic potential, including two samples (3728T and 3754T) from our previous mixed BF^+^ colorectal cancer slurry studies (5). Individual BF^+^ patient slurries induced variable tumorigenesis, with the 3728T slurry inducing robust tumorigenesis in both GF *Apc^Min/+^* (Fig. 1A; *P* = 0.001, 3728T vs. sham) and specific pathogen–free (SPF) *Apc^Min/+^* mice (Supplementary Fig. S1A; *P* = 0.042, 3728T vs. sham). Similar variability was observed with BF^−^ patient slurries, with only the 3752T patient slurry inducing tumors (Fig. 1B;*P* < 0.001, 3752T vs. sham).

16S rRNA amplicon sequencing of the individual BF^+^ and BF^−^ slurries as well as stools from slurry-gavaged GF *Apc^Min/+^* mice revealed mostly commensal populations dominated by *Bacteroides, Blautia*, and *Clostridium* spp. (Fig. 1C). To further evaluate which microbes contributed to tumorigenesis, we extensively cultured distal colonic tissues from 3728T slurry–gavaged GF *Apc^Min/+^* mice euthanized at various time points [4, 7, 14, 28, and 56 days post-inoculation (p.i.)]. Species identification with matrix-assisted laser desorption ionization time-of-flight mass spectrometry (MALDI-TOF MS) revealed 34 unique species (Supplementary Table S1); 30 of these isolates were successfully propagated from frozen stocks and combined into a single inoculum (3728T isolates, see Methods). This 3728T isolate mixture recapitulated the distal colonic tumorigenesis of the original 3728T slurry in GF *Apc^Min/+^* mice (Fig. 1D; *P* < 0.0001, 3728T isolates vs. sham).

### *C. difficile* Drives Tumorigenesis of the 3728T Isolates

Notably, the 3728T isolate mixture did not contain previously established tumorigenic bacteria such as ETBF, pks^+^Ec, or *Fusobacterium nucleatum* (Supplementary Table S1). However, toxigenic strains of the Gram-positive, spore-forming anaerobic pathogen *C. difficile* were isolated from all mice gavaged with the 3728T slurry (Supplementary Table S1). Reanalysis of our previously published 16S rRNA amplicon sequencing data (12) demonstrated that *C. difficile* was enriched in the stools and colonic tissue of mice gavaged with the tumorigenic mixed patient BF^+^ colorectal cancer slurries at both acute (1 week p.i.) and chronic (12 weeks p.i.) time points, but not in the mice gavaged with the nontumorigenic BF^−^ healthy biopsy tissues (Supplementary Fig. S1B). Furthermore, prior RNA sequencing (RNA-seq) demonstrated that expression of the *C. difficile* toxins *tcdA* and *tcdB* was enriched in the BF^+^ colorectal cancer slurry-gavaged mouse colons compared with BF^−^ tissue gavaged mice (12). Thus, we hypothesized that toxigenic (TcdA^+^TcdB^+^) *C. difficile* could be driving murine tumorigenesis. 16S rRNA amplicon sequencing combined with qPCR testing for *tcdA* and *tcdB* of the individual patient BF^+^ and BF^−^ colorectal cancer slurries from the present study as well as stools from mice gavaged with these slurries revealed that toxigenic *C. difficile* (*tcdA^+^, tcdB^+^*) was more abundant in the protumorigenic slurries (3728T and 3752T) and their respective mouse stools compared with all other slurries (Supplementary Fig. S1C–S1F). The relative abundance in terminal mouse stools correlated with colonic tumor number in the mice [Supplementary Fig. S1G and S1H; *P* = 0.002 *C. difficile* relative abundance in mouse stools vs. colonic tumor counts for BF^+^ tumor (BF^+^T) slurries; *P* < 0.0001 for BF^−^ tumor (BF^−^T) slurries]. In contrast, nontumorigenic slurries displayed low levels of *C. difficile* (>Ct 35) and did not have detectable *tcdA* or *tcdB* (Supplementary Fig S1C–S1F). We successfully cultivated a *C. difficile* strain, named CIm2663_BF^+^T (clinical isolate from GF mouse 2663 gavaged with a BF^+^T mixed patient slurry), from frozen colonic tissue from a mouse previously gavaged with the mixed BF^+^ colorectal cancer slurry, which included the 3728T slurry (12). We compared this strain to one of the toxigenic *C. difficile* strains isolated from a mouse gavaged with the 3728T slurry (strain CIm161_3728T, from GF mouse 161 gavaged with the 3728T slurry) by whole-genome sequencing. These toxigenic *C. difficile* strains, CIm2663_BF^+^T and CIm161_3728T, were >99.98% identical and were therefore jointly renamed CIm_3728T, reflecting their common origin from patient 3728T (Supplementary Fig. S1I; Supplementary Table S2). A *C. difficile* strain was also cultured from GF mice gavaged with the 3752T slurry (CIm_3752T). Whole-genome sequencing, qPCR testing, and *in vitro* cytotoxicity assays of the individual *C. difficile* strains demonstrated that both CIm_3728T and CIm_3752T harbored *tcdA* and *tcdB* but were of two different ribotypes, FP485 and 014-020, respectively (Supplementary Table S2; Supplementary Fig. S1I). Neither strain contained binary toxin (*cdt*), consistent with the phylogeny of these two ribotypes.

To further test the tumorigenic potential of *C. difficile*, we compared toxigenic and nontoxigenic strains in an SPF *Apc^Min/+^* mouse model developed herein (see Methods). Both the toxigenic ATCC strain 9689 and CIm_3728T induced distal colonic tumors in SPF *Apc^Min/+^* mice, whereas the nontoxigenic *C. difficile* ATCC strain 700057 did not (Fig. 1E and F). Collectively, these results indicated that toxigenic, but not nontoxigenic, *C. difficile* are procarcinogenic.

We next tested the relationship between *C. difficile in vivo* toxin production and tumorigenesis in both GF and SPF murine models. Stools from GF *Apc^Min/+^* mice gavaged with the 3728T isolates plus CIm_3728T or CIm_3752T displayed chronic elevations in toxin levels at all time points, but toxin levels at intermediate (4 weeks) or late (10 weeks) time points did not correlate with tumorigenesis (Supplementary Fig. S2A–S2D). Similarly, in SPF *Apc^Min/+^* mice colonized with CIm_3728T or the toxigenic ATCC strain, longitudinal fecal sampling revealed stable *C. difficile* colonization with persistent *C. difficile* toxin production for 12 weeks (Supplementary Fig. S2E and S2F). Of note, *C. difficile* colonization and toxin production were significantly higher at 4 weeks p.i. with the CIm_3728T strain compared with the ATCC toxigenic 9689 strain [Supplementary Fig. S2E and S2F; *P* = 0.030 for colony-forming units (CFU), *P* = 0.004 for toxin production]. Final colonic tumor counts at 12 weeks p.i. correlated with toxin amounts detected at this 4-week time point, but not at other time points (Supplementary Fig. S2G). In our SPF *Apc^Min/+^* model, we also tested the tumorigenicity of the 630*∆erm* strain of *C. difficile*, which is toxigenic but less virulent than currently circulating epidemic *C. difficile* strains (13, 14). Strain 630*∆erm* did not significantly enhance tumor formation compared with sham mice (Supplementary Fig. S2H). Fecal toxin levels with 630*∆erm* were detected only at 1 week p.i. despite persistent stable colonization (Supplementary Fig. S2I and S2J). Together, these data suggest chronic toxin production plays an important role in tumor induction.

To determine whether *C. difficile* was required for tumorigenesis of the 3728T isolates, we gavaged GF *Apc^Min/+^* mice with the 3728T isolate mixture lacking *C. difficile*. These 29 isolates failed to induce tumorigenesis at 10 weeks p.i. (Fig. 1G), whereas the inclusion of the CIm_3728T or CIm_3752T strain in the isolate mixture significantly induced colonic tumors (Fig. 1G; *P* = 0.001 and *P* = 0.01 compared with shams, respectively). Representative methylene blue–stained colons demonstrated that these *C. difficile*–associated tumors localized primarily to the distal 2 to 3 cm of the colon (Fig. 1H), with frequent rectal prolapse observed (8/24 mice with *C. difficile* vs. 0/13 shams or 3728T isolates without *C. difficile*, Fisher *P* = 0.032). Small intestinal tumorigenesis was not altered by *C. difficile* (Supplementary Fig. S2K and S2L), and no mouse sex differences in colonic tumor formation were observed (Supplementary Fig. S2M and S2N).

To further test the ability of *C. difficile* to drive tumorigenesis, we added the *C. difficile* CIm_3728T strain to the nontumorigenic colorectal cancer slurry 3979T, again yielding distal colonic tumors (Fig. 1I; *P* = 0.026, 3979T slurry + CIm_3728T vs. shams). *C. difficile* was highly lethal in monocolonized GF *Apc^Min/+^* mice but did induce distal colonic tumors in the mice that survived (Supplementary Fig. S2O; *P* = 0.029). In combination with our SPF *Apc^Min/+^* data (Fig 1E), these results suggest that *C. difficile* is necessary and sufficient for driving tumorigenesis in the context of these diverse microbial environments.

### *C. difficile*–Induced Tumorigenesis Is Associated with Altered Mucosal Immune and Epithelial Cell Transcriptional Responses

**Figure 2.**
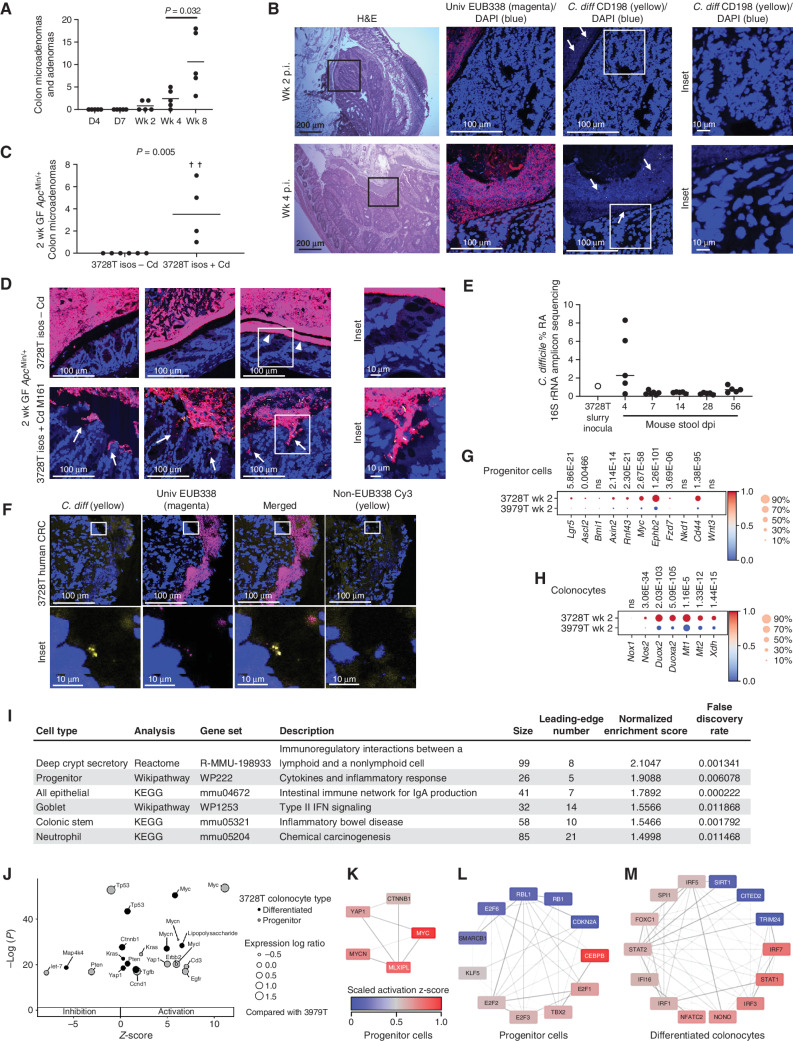
*C. difficile* and the 3728T slurry induce rapid tumorigenesis in GF *Apc^Min/+^* mice despite representing only a fraction of the luminal and mucosal microbiota. **A** and **B,** Twenty-five GF *Apc^Min/+^* mice were gavaged with the 3728T slurry; five mice were sacrificed per time point. **A,** Colonic microadenoma and adenoma counts. **B,** Hematoxylin and eosin (H&E) staining of a representative microadenoma is shown from a mouse at 2 weeks p.i. (top), whereas a large adenoma coated by a dense BF is shown from a mouse at 4 weeks p.i. (bottom). Confocal imaging of Carnoy's-fixed mouse distal colons stained with the EUB338 universal probe (magenta), Cd198 *C. difficile* probe (yellow), or DAPI counterstain (blue). White arrows highlight individual *C. difficile* rods. **C** and **D**, Microadenomas (**C**) and FISH staining (**D**) of 2 weeks p.i. distal colons from GF *Apc^Min/+^* mice gavaged with the 3728T isolates ± *C. difficile* CIm_3728T. Crosses represent mouse deaths. *n* = 6 mice per group, from two independent experiments. FISH images represent three separate mice per inoculum. White arrowheads denote areas with occasional mucus-invasive BFs directly interacting with epithelium in mice gavaged with the 3728T isolates without *C. difficile* (3728T isos – Cd). White arrows (bottom) represent areas with crypt-invasive BFs in the mice gavaged with the 3728T isolates + *C. difficile*. Statistical significance in **A** and **C** was calculated via Kruskal–Wallis followed by Mann–Whitney tests. Mann–Whitney *P* values are shown. **E,** Relative abundance (RA) of *C. difficile* derived from 16S rRNA amplicon sequencing of the original human slurry (3728T) or mouse stool obtained at sacrifice of mice in the time course experiment following gavage with the 3728T slurry. dpi, days post-inoculation. **F,** FISH of the original patient tumor 3728T. CRC, colorectal cancer. **G–M,** scRNA-seq and gene set enrichment analysis of 3728T slurry (*n* = 3)– versus 3979T slurry (*n* = 3)–gavaged GF *Apc^Min/+^* mice at 2 weeks p.i. Heat maps depict genes enriched in canonical Wnt (**G**) or ROS (**H**) signaling pathways, with Wilcoxon rank-sum test *P* values above each gene. **I,** Inflammatory signaling pathways enriched in specific cell types from 3728T slurry– versus 3979T slurry–gavaged mice at 2 weeks p.i. KEGG, Kyoto Encyclopedia of Genes and Genomes. **J,** Inhibition and activation of the most significant upstream regulators in either progenitor or differentiated colonocytes. Expression log ratio is an effect size statistic. **K** and **L,** Network analysis of transcription factors from IPA upstream regulators enriched in the progenitor cell gene list revealed activation of Wnt/Myc signaling and proliferation, respectively. Lines connect transcription factors with shared target genes in the gene list. Darker lines indicate a greater number of shared target genes. **M,** Similar network analysis for differentiated colonocytes showed activation of the innate immune response.

Our prior work suggested that early mucosal responses to protumorigenic bacteria are critical for colonic tumor induction (15–17). Thus, to define early tumorigenic events associated with the *C. difficile*–positive 3728T slurry, we first examined the histologic appearance of microadenomas in colons from 3728T slurry–gavaged mice at various time points. A small number of dysplastic crypts (precursors to microadenomas) were visible in some mice at day 4 p.i. (Supplementary Fig. S3A). Microadenomas formed as early as 14 days p.i. and progressively increased over time (Fig. 2A and B; *P* = 0.032 4 weeks vs. 8 weeks p.i. by Mann–Whitney). A distal colonic adenoma (2.4 × 1.5 mm wide), coated with a dense BF, was observed in one mouse at day 28 p.i. (Fig. 2B, bottom). Similarly, inoculation with the 3728T isolates, including CIm_3728T, also yielded microadenomas by day 14 p.i. (Fig. 2C; *P* = 0.005, 3728T isolates + Cd vs. 3728T isolates − Cd). Mucus-invasive BFs were detected in focal regions of the distal colons in five of six mice gavaged with the 3728T isolates lacking *C. difficile*, but the presence of *C. difficile* triggered a widespread, dramatic crypt invasion of these BFs in the distal colon of each mouse (Fig. 2D). Given the rapid *C. difficile*–induced tumorigenesis in both the 3728T slurry– and 3728T isolate–gavaged mice, we anticipated that *C. difficile* would be a dominant member of the microbiota in the stools. However, similar to the original 3728T patient slurry as well as mice analyzed at 14 weeks p.i. in Supplementary Fig. S1F, fecal 16S rRNA amplicon sequencing at early time points revealed *C. difficile* at <2% relative abundance in most mice, with the exception of day 4 p.i. (*C. difficile* relative abundance range, 0.27%–8.30% at day 4 p.i.; Fig. 2E). Using a *C. difficile*–specific probe, fluorescence *in situ* hybridization (FISH) analysis of these mouse tissues demonstrated that *C. difficile* comprised only a minority of the mucosal BF in these gnotobiotic mice (Fig. 2B and D). Analysis of the original 3728T patient tumor also detected *C. difficile* at low abundance by FISH in the tumor BF (Fig. 2F). However, BFs were not required for *C. difficile*–associated tumorigenesis, as in our SPF model these BFs were not present, although periodic mucosal colonization was observed (Supplementary Fig. S3B). Additionally, unlike tumor-homing microbes such as *F. nucleatum* (18), *C. difficile* did not preferentially localize to microadenomas or tumors compared with the surrounding normal tissue in either our GF or SPF *Apc^Min/+^* model (Supplementary Fig. S3C and S3D).

Prior data have shown that the antibiotic clearance of the procarcinogenic organism ETBF at 5 days or 2 weeks p.i. prevented colonic tumor formation in SPF *Apc^Min/+^*mice, with earlier clearance demonstrating stronger prevention (15). Similarly, the use of vancomycin beginning at 1 week p.i. to clear *C. difficile* in GF *Apc^Min/+^* mice gavaged with the 3728T isolates +Cd led to a near-complete blockade of microadenoma formation at 2 weeks p.i. (Supplementary Fig. S3E; *P* = 0.011). These results, combined with results in Supplementary Fig S2A–S2J, suggest that persistent colonization and toxin production over the course of several weeks, rather than *C. difficile* microbiota dominance, are likely critical to promoting tumorigenesis, whereas invasive BFs may impact but are not essential for *C. difficile* tumorigenesis.

We next examined whether early colonic transcriptional responses in epithelial subsets could elucidate how the *C. difficile*–positive 3728T slurry was tumorigenic, whereas others negative for *C. difficile*, such as the 3979T slurry, were not tumorigenic. We thus performed single-cell RNA-seq (scRNA-seq) of distal colons from GF *Apc^Min/+^* mice inoculated with either the tumorigenic 3728T slurry or the nontumorigenic 3979T slurry at 2 weeks p.i. These scRNA-seq data revealed differential clustering based on 3728T versus 3979T slurry inoculation (Supplementary Fig. S3F and S3G). Differential gene expression analysis showed that the 3728T slurry–gavaged mice displayed the induction of two critical procarcinogenic signatures compared with the 3979T slurry: (i) canonical Wnt signaling in progenitor cells (Fig. 2G) and (ii) and reactive oxygen species (ROS) production in differentiated colonocytes (Fig. 2H). Wnt signaling is a hallmark of both sporadic and hereditary colorectal cancer (19), whereas oxidative stress has been previously implicated in multiple microbially mediated colorectal cancer mouse models (9, 20). Upregulation of *Duox2* has also specifically been linked to *C. difficile* infection with *tcdB*^+^ strains (21). Gene set enrichment analysis using WebGestalt (22) demonstrated that multiple inflammatory-associated signaling pathways were induced in 3728T slurry–gavaged mice (Fig. 2I; Supplementary Fig. S3H), further suggesting that the 3728T slurry accelerates tumorigenesis through procarcinogenic signaling and inflammation.

We additionally used Ingenuity Pathway Analysis (IPA; https://digitalinsights.qiagen.com) to compare differential gene expression. This enrichment analysis from both progenitor and differentiated colonocytes predicted upstream regulators specifically implicated in inhibition or activation of the target genes detected (Supplementary Table S3). Among the 50 regulators with the lowest *P* values in each cell type in 3728T slurry–inoculated mice compared with the 3979T slurry, specific upstream regulators of interest were plotted with their predicted activation state or z-score (Fig. 2J). For example, the oncogene *Myc* was markedly activated in progenitor cells but less so in differentiated cells, whereas the tumor suppressor genes *p53* and *Pten* were more inhibited (lower z-score) in progenitor cells than in differentiated cells (Fig. 2J). Next, we selected all the transcription factors from the IPA-generated list of upstream regulators to identify activated signaling pathways with shared target genes (Fig. 2K–M). In progenitor colonocytes, we found activation of the Wnt/Myc/Hippo signaling pathways (Fig. 2K) as well as the maintenance of stemness (Klf5), proliferation (E2f family), inflammatory response (Cebpb), and inhibition of Pten (Tbx2; Fig. 2L). The same analysis was performed for the gene list from differentiated colonocytes, which showed increased activation of innate inflammatory response signaling pathways, as evidenced by high z-scores in the Stat and Irf families (Fig. 2M). Together, these observations support the hypothesis that the upregulation of oncogenes and downregulation of tumor suppressors along with procarcinogenic inflammation promote tumor development in our model.

**Figure 3.**
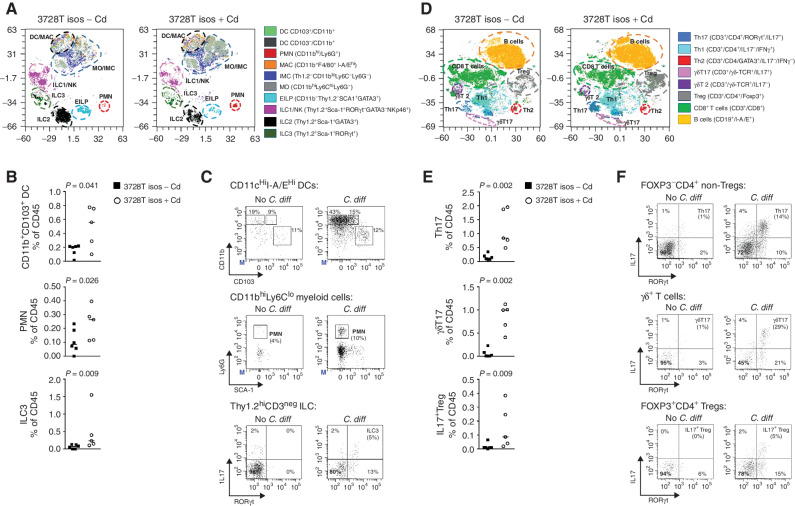
*C. difficile* induces the activation of myeloid cells and IL17-producing lymphoid subsets. Multiparameter flow cytometry analysis of colonic lamina propria innate (myeloid and ILCs) and adaptive immune cell populations of LPL isolated from colons collected at 2 weeks p.i. from GF *Apc^Min/+^* mice gavaged with the 3728T isolates with (*n* = 5) or without (*n* = 6) *C. difficile* strain CIm_3728T. Results from two independent experiments. **A,** Opt-SNE projection of myeloid and ILC populations. **B,** Dot plots of myeloid cell type abundance as a percentage of the CD45 population. **C,** Representative gating of DCs, PMN, and IL17 expression by ILC3 cells. **D,** Opt-SNE projection of adaptive populations (B and T lymphocytes). **E,** Dot plots of changes in abundance of lymphoid populations with *C. difficile*. **F,** Representative gating of lymphoid subsets. Opt-SNE projections are used for visualization purposes only. Statistical analyses in **B** and **E** represent Mann–Whitney *t* tests based on the gating in **C** and **F**, respectively. DC, dendritic cells; EILP, early innate lymphoid progenitors; ILC, innate lymphoid cells; IMC, innate myeloid cells; LPL, lamina propria leukocytes; MAC, macrophages; MO, monocytic cells; NK, natural killer; PMN, polymorphonuclear cells; Treg, regulatory T cells.

To determine whether *C. difficile*–induced protumorigenic signaling extended to the immune cell compartment, we performed high-dimensional flow cytometry on colonic lamina propria leukocytes (LPL) isolated from GF *Apc^Min/+^* mice gavaged with the 3728T isolate mixture with or without *C. difficile* (CIm_3728T strain). Optimized t-distributed stochastic neighbor embedding (Opt-SNE) visualization and standard gating of the flow cytometry data showed that the presence of the isolates with *C. difficile* triggered a significant increase in the abundance and activation (Fig. 3A–C) of myeloid cells and innate lymphoid cells (ILC), including CD11b^+^CD103^+^ dendritic cells (*P* = 0.041), polymorphonuclear (PMN) leukocytes (encompassing neutrophils, eosinophils, and basophils; *P* = 0.026), and type III ILCs (ILC3; *P* = 0.009) in mouse distal colons at 2 weeks p.i. IHC staining of 3728T slurry–gavaged mice demonstrated that an early enrichment of Ly-6G^+^ granulocytic infiltrates at day 4 p.i. in the distal colons shifted toward a mature, activated F4/80^+^ macrophage phenotype later in infection (day 28 p.i.) compared with sham mice (Supplementary Fig. S4A–S4C). The accumulation of PMNs triggered by the 3728T isolates with *C. difficile* was associated with a potent type 3 mucosal immune response (Fig. 3D–F) marked by the enrichment of IL17-producing subsets, including Th17 cells (*P* = 0.002), γδT17 cells (*P* = 0.002), IL17^+^ regulatory T cells (*P* = 0.009), and the aforementioned ILC3s (Fig. 3B), in the high-parameter flow cytometry analyses.

### TcdB Is Required for *C. difficile*–Induced Tumorigenesis

**Figure 4.**
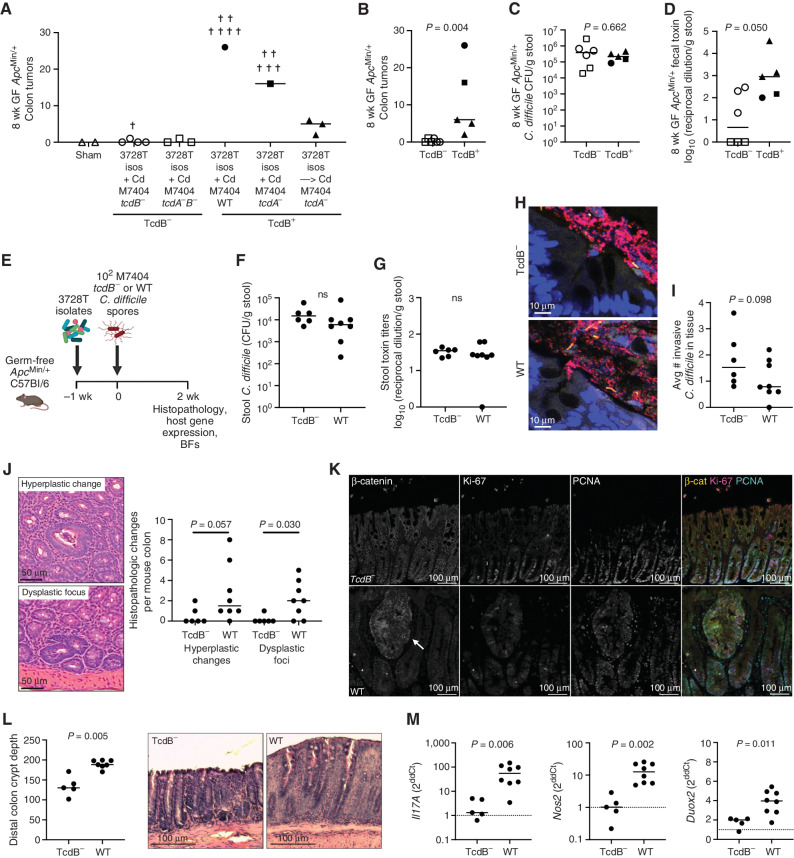
*C. difficile*–associated tumorigenesis is toxin B (TcdB)–dependent. **A,** GF *Apc^Min/+^* mice were gavaged with WT or isogenic *tcdA^−^*, *tcdB^−^*, or *tcdA^−^tcdB^−^* TargeTron mutant strains of M7404, an epidemic strain of ribotype 027, either simultaneously with the 3728T isolates or 1 week later. Tumor counts at 8 weeks p.i. were analyzed. Crosses represent mouse deaths. *n* = 2–7 mice per group, from two independent experiments. **B,** Summary graph showing colonic tumor induction of all TcdB^−^ versus TcdB^+^ M7404 strains in combination with the 3728T isolates in GF *Apc^Min/+^* mice. **C** and **D,***C. difficile* stool CFUs and Vero toxin titers. Mann–Whitney *P* value is shown. **E–M,** Experimental design for 2-week experiments with M7404 strains: mice were gavaged with the 3728T isolates − Cd 1 week prior to *C. difficile* gavage with either the *tcdB^−^* (*n* = 6 mice) or WT M7404 strain (*n* = 8 mice). **F** and **G,***C. difficile* stool CFUs and toxin titers. **H,** Representative crypts with BF invasion, stained in red for EUB338 and yellow for Cd198. **I,***C. difficile* tissue invasion. **J,** Representative H&E images and quantitation of histopathologic changes at 2 weeks p.i. **K,** Representative images of β-catenin, Ki-67, and PCNA immunofluorescent staining of distal colonic epithelium in M7404 TcdB^−^ (top)– versus M7404 WT (bottom)–inoculated mice. Arrow denotes a dysplastic focus in the M7404 WT–inoculated mouse. **L,** Quantitation and representative images of crypt depth in the distal colon. **M,** Host gene expression in colon. Dashed line represents 3728T isolates − Cd-negative control. Statistics shown are Mann–Whitney *t* tests. ns, not significant.

Given that multiple toxigenic strains (CIm_3728T, CIm_3752T, and ATCC 9689) were tumorigenic in our mouse models, we tested whether TcdA and/or TcdB were essential to tumor induction. TcdB is considered the more critical toxin in *C. difficile* infection given that *tcdA^−^tcdB^+^* strains replicate symptomatic infection in both patients and animal models (21, 23). We thus tested isogenic mutant strains of the M7404 (ribotype 027) *C. difficile* isolate, in which the *tcdA* and/or *tcdB* gene has been inactivated via insertion of a lincomycin cassette (21). The M7404 wild-type (WT) and *tcdA*-mutant (*tcdA^−^*) strains resulted in rapid weight loss and were highly lethal in GF *Apc^Min/+^* mice even in combination with the 3728T isolates (Fig. 4A; Supplementary Fig. S4D). Gavaging the 3728T isolates 1 week prior to *C. difficile* resulted in improved survival outcomes while maintaining tumor induction (Fig. 4A; Supplementary Fig. S4D). Collectively, the mice that survived colonization with *tcdB^+^* strains of M7404 developed significantly more colonic tumors compared with mice gavaged with *tcdA^+^tcdB^−^* or *tcdA^−^tcdB^−^* strains (Fig. 4B; *P* = 0.004, *tcdB^−^* vs. *tcdB^+^*). The presence of *tcdB* did not affect *C. difficile* colonization levels (Fig. 4C), though *tcdB^−^ and tcdA^−^B^−^* strains produced significantly less toxin as measured in terminal stool samples, largely driven by the complete lack of toxin production in the *tcdA^−^tcdB^−^* mice (Fig. 4D).

Using the sequential gavage of the 3728T isolates followed by either the *tcdB^−^* or WT M7404 strains (Fig. 4E), we tested the impact of TcdB on early tumorigenic mechanisms at 2 weeks p.i. Stool CFUs and toxin titers were not significantly different between *tcdB^−^* (due to TcdA production) and WT strains (Fig. 4F and G). Crypt-invasive BFs could be observed in colons from both the *tcdB^−^* and WT M7404 mouse colons (Fig 4H), and *C. difficile* tissue invasion was also not significantly different (Fig. 4I). However, *tcdB^−^* M7404 significantly blunted the formation of early hyperplasia (*P* = 0.057) and dysplastic foci (*P* = 0.030) compared with WT M7404 (Fig. 4J). Further, WT M7404–gavaged mice displayed several hallmarks of increased Wnt/β-catenin signaling and inflammation compared with the *tcdB^−^* strain, including increased β-catenin, Ki-67, and peripheral cell nuclear antigen (PCNA) staining in dysplastic crypts (Fig. 4K), as well as elongated crypts in the distal colon (Fig. 4L; *P* = 0.005). WT M7404 was also associated with elevated expression of *Il17A* and the oxidative stress markers *Nos2* and *Duox2* in distal colons of these mice compared with the *tcdB^−^* strain (Fig 4M; *P* = 0.006, *P* = 0.002, and *P* = 0.011, respectively), further demonstrating that TcdB is critical for the induction of multiple procarcinogenic mechanisms in both epithelial cells and mucosal immune responses.

## DISCUSSION

Overall, our data implicate toxigenic *C. difficile* as a previously unrecognized driver of colonic tumorigenesis under multiple microbial environments. Toxigenic *C. difficile* was associated with the tumorigenesis of both BF^+^ (3728T) and BF^−^ (3752T) patient tumor slurries and was necessary and sufficient to drive the tumorigenesis of the 3728T isolates, convert the nontumorigenic 3979T slurry into a protumorigenic one, and drive tumorigenesis in an SPF model. Although initially discovered in our colorectal cancer patient population, this tumorigenic potential was also evident in clinical toxigenic strains derived from patients with *C. difficile* infection but not in the attenuated 630 strain. Our results suggest that prolonged mucosal exposure to TcdB *in vivo* is essential and that *C. difficile* and its toxins may continue to promote tumorigenesis over time, as microadenomas substantially increased from 4 to 8 weeks p.i. with the 3728T slurry (Fig. 2A), whereas clearance with vancomycin at 1 week p.i. blunted microadenoma formation triggered by the 3728T isolates with *C. difficile* (Supplementary Fig. S3E). These observations raise particular concern about *C. difficile*–associated colorectal cancer in those with recurrent *C. difficile* infections or inflammatory bowel disease, the latter of whom are at a greater risk for both *C. difficile* infection and colorectal cancer. At present, *C. difficile* is considered an opportunistic pathogen during cancer treatment, including colorectal cancer, with only scant data suggesting linkage of *C. difficile* with colorectal cancer (Supplementary Fig. S4E; refs. 24–27). Future work identifying the effect of *C. difficile* colonization, duration, and toxin production on epithelial–immune interactions in humans will be needed to determine the tumorigenic risk to patients.

Mechanistically, our data point to multiple mechanisms of *C. difficile*–induced tumorigenesis, including increased Wnt/β-catenin signaling, ROS production, and a procarcinogenic mucosal immune response characterized by IL17 production from multiple cellular sources as well as a prominent myeloid infiltration. Although nontoxigenic strains or isogenic mutants of *tcdB* demonstrated the requirement of TcdB for *C. difficile*–associated tumorigenesis, *C. difficile* CFUs and toxin titers did not consistently predict tumor burden, suggesting that variability in host responses and/or other members of the microbiota are likely strong modifiers. The upregulation of oncogenes and downregulation of tumor suppressors in the progenitor epithelial compartment highlights the potential importance of spatial heterogeneity in driving *C. difficile*–associated tumorigenesis. Notably, our *in vivo* Wnt/β-catenin data in crypt progenitor cells are in contrast with *in vitro* studies that have suggested purified TcdA and TcdB may inhibit Wnt/β-catenin signaling (28, 29). Others have also shown that TcdB is required for *C. difficile*–associated dysregulation of mouse colonic stem cell function during acute infection (29–31). These data suggest that nuances in the spatial localization of host responses may be critical for tumor biology. Our FISH analyses revealed that *C. difficile*, particularly in combination with the 3728T isolates, formed highly invasive BFs in GF *Apc^Min/+^* mice, reaching deep into colonic crypts; however, these BFs were neither required for *C. difficile*-mediated tumorigenesis nor dependent on TcdB production. Nevertheless, these crypt-invasive BFs may facilitate interactions between *C. difficile* and multiple colorectal cancer precursor cell types. Additionally, although our data support a role for *C. difficile* as a driver of tumorigenesis, this does not preclude the possibility that other BF communities with distinct compositions may still be protumorigenic.

Overall, our results in tumor-susceptible mouse models combined with limited prior human studies introduce *C. difficile* as a plausible promoter of human colorectal cancer (24–27). Translation to human studies will be imperative to understand the epidemiology, mucosal biology, and immunology of persistent *C. difficile* colonization and its relationship to colitis-associated and sporadic colorectal cancer, including early-onset colorectal cancer.

## METHODS

### Patient Colorectal Cancer Slurries (BF^+^ and BF^−^)

Excess colorectal cancer tissue samples not needed for pathology were obtained from colorectal cancer surgical specimens from patients undergoing surgical resection at Johns Hopkins Hospital. Specimens were either flash-frozen or fixed in Carnoy's and evaluated for mucus-invasive BFs as previously described (10). Punch biopsies (3 mm) taken from mucosal regions were weighed, homogenized in 50 μL sterile prereduced 1× PBS, and diluted 1/20 weight/volume (w/v) in additional sterile prereduced PBS in an anaerobic chamber. For gavage into mice, these working stocks were diluted an additional 1/10 for a total of 1/200 w/v.

### Ethics Reporting

This study was approved by the Johns Hopkins Institutional Review Board (IRB). The six colorectal cancer samples utilized in this study were obtained with written informed consent, and, in addition, tissue studies were conducted under an IRB-approved excess tissue collection protocol. All procedures were in accordance with the Health Insurance Portability and Accountability Act and the Belmont Report.

### Animals

GF and SPF C57BL/6 *Apc^Min/+^* mice of the ∆716 *Apc* genotype were maintained in accordance with protocols approved by the Johns Hopkins University Animal Care and Use Committee (protocols: MO20M85 and SP20M233).

GF *Apc^Min/+^* mice were housed in the Germ-Free Mouse Core in the Johns Hopkins University School of Public Health in sterile bubble isolators (Controlled Environment Products #041-4205-0888) with an unlimited sterile diet (LabDiet JL rat and mouse/Auto 6F 5k67) and double-autoclaved tap water. Fecal pellets from GF mice were screened monthly by culture and qPCR to ensure maintenance of their GF status. For experiments, 6- to 12-week-old mice were transferred into sterile isocages (Allentown, Sentry SPP) with both rack and intracage HEPA filters (#HF884-H14 and #228535). GF *Apc^Min/+^* mice 6 to 12 weeks of age were gavaged with 100 μL of either the diluted patient colorectal cancer slurries or the 3728T bacterial isolates ± 10^4^ spores of the human colorectal cancer–derived *C. difficile* strains or 10^2^–10^4^ spores of the epidemic *C. difficile* strain M7404 (see Supplementary Methods for additional details on spore preparation). For experiments involving the highly pathogenic M7404 *C. difficile* strains, all mouse groups were also given a water gel pack during the acute inoculation period to assist in recovery from acute *C. difficile* illness. For the gnotobiotic vancomycin clearance experiment, a fresh solution of 0.4 mg/mL vancomycin was sterile-filtered, and then transferred to sterile water bottles and given to gnotobiotic mice beginning at 1 week p.i. The solution was replaced twice a week. *C. difficile* fecal colonization was quantified from frozen stools diluted in a 10-fold dilution series in sterile, prereduced PBS. Five microliters of each dilution were spotted onto cycloserine–cefoxitin fructose plates supplemented with 0.1% taurocholate (TCCFA). CFUs were enumerated after 24 hours of growth in an anaerobic chamber.

SPF *Apc^Min/+^* mice were housed and bred with autoclaved food, bedding, and chlorinated water in the SPF facility in the Cancer Research Building II at Johns Hopkins University. For SPF *Apc^Min/+^* experiments with BF^+^ slurries, 6- to 10-week-old mice were gavaged with 100 μL of cefoxitin (500 μg/mL) 3 days prior to inoculation and given cefoxitin (500 mg/L) in their drinking water for 2 days. Following a 1-day wash-out period, mice were gavaged with 100 μL of either the 3728T slurry or the 3754T slurry. No further antibiotics were given. For SPF *Apc^Min/+^* experiments with toxigenic/nontoxigenic *C. difficile* strains, 6- to 10-week-old male and female mice were given vancomycin (Sigma; 0.05 mg/mL) and gentamicin (Sigma; 0.035 mg/mL) in drinking water for 5 to 7 days, followed by 2 to 3 days of regular drinking water prior to the inoculation of 10^6^*C. difficile* spores in PBS (or PBS alone for sham mice) via single oral gavage. Gentamicin (0.035 mg/mL) supplement in the drinking water was resumed after inoculation and continued for the duration of the 12-week experiment. *C. difficile* fecal colonization was quantified by qPCR with 100 ng stool DNA and/or by plating serial dilutions of heat-treated mouse stool homogenates (60°C for 1–2 hours, which kills vegetative cells but not *C. difficile* spores) on CCFA-HT or BHIS-T [brain heart infusion (BHI) agar supplemented with *C. difficile* supplement (Sigma) and 0.1% taurocholate] plates.

GF and SPF mice were monitored for body weight loss, hunched posture, and other humane endpoints; otherwise, mice were sacrificed at their predetermined endpoint (day 4–week 14 p.i.). All experimental groups were matched by age, sex, and litter. Gross colonic tumors from mice at 8 to 14 weeks p.i. were counted under a dissecting scope (Leica ES2 Stereo Microscope) following methylene blue staining of flushed and opened formalin-fixed colons. Microadenomas were enumerated by a pathologist via hematoxylin and eosin scoring of tissue sections; a microadenoma was defined as a small adenoma with at least 3 dysplastic crypts.

### Isolation of Bacteria from 3728T-Gavaged Mice (Culturomics)

GF *Apc^Min/+^* mice were gavaged with the 3728T slurry as described above. Distal colon biopsies (approximately 2.5–3 cm from the rectum) from two mice per time point (days 4, 7, 14, 28, and 56 p.i.) were excised for culture. Biopsies were washed 5× with sterile 1× PBS and then homogenized with a pestle in 50 μL sterile, prereduced water in an anaerobic chamber. All washes were reserved and concentrated by spinning down for 5 minutes at 10,000 × *g* and resuspended in 1 mL sterile PBS. Both the firmly adherent microbes from the distal colon (15 μL per plate) as well as the loosely adherent microbes from the concentrated washes (10 μL per plate serially diluted) were cultured using multiple media. Aerobic media included (i) aerobic tryptic soy blood agar (TSBA; made in-house) and (ii) chopped meat medium with glucose (CMG; Anaerobe Systems). Anaerobic media included brucella blood agar (BRU; Anaerobe Systems), phenylethyl alcohol blood agar (PEA; Anaerobe Systems), Bacteroides bile esculin agar (in-house), BHI (in-house), and CMG (Anaerobe Systems). Colonies were grown 24 to 72 hours at 37°C. All distinct morphologies on each agar plate were chosen for follow-up identification via MALDI-TOF MS (Bruker) in the Johns Hopkins Clinical Microbiology Laboratory, resulting in approximately 3 to 10 colonies chosen per plate. In total, several hundred colonies were screened. Thirty-five unique species were identified and frozen back in 20% prereduced milk at −80°C. Several isolates were identified by 16S rRNA amplicon sequencing instead as MALDI-TOF MS yielded “no ID” messages. Additionally, MALDI-TOF MS cannot distinguish between the closely related *Bacteroides vulgatus* and *Bacteroides dorei*; 16S rRNA amplicon sequencing confirmed this 3728T isolate to be *Bacteroides dorei*.

### Creation of 3728T Isolate Mixture

Isolates obtained above from the 3728T-gavaged mice were subsequently restreaked from their respective frozen milk stocks individually, and species identification was confirmed a second time by MALDI-TOF MS. A single colony of each bacterium was then subcultured individually into separate 3 mL broth cultures for 24 to 72 hours depending on the growth rate or in the case of the spore-forming Clostridia (with the exception of *C. difficile*; see below) onto a BHI plate 7 days in advance to allow for sporulation. Optimal growth conditions were as follows: the aerobic strains *Bacillus circulans* and *Micrococcus luteus* were grown aerobically on BHI for 24 to 48 hours. The anaerobic strains *Gordonibacter pamelaeae, Blautia coccoides*, and *Flavonifracter plautii* were grown anaerobically on blood agar plates (either TSBA or BRU) for 48 to 72 hours. All other strains were grown anaerobically on BHI plates for 24 to 48 hours. A crude mixture of the isolates was generated by combining 1 mL of each liquid growth with four streaks of each plate of the spore-forming Clostridia into a 50-mL conical, washing 1× with PBS, and resuspending in 5 mL of 20% prereduced skim milk in water. The 3728T isolate mixture was then aliquoted and stored at −80°C.

### FISH

FISH was performed on paraffin-embedded Carnoy's- or poloxamer-fixed tissues with probes targeting the *16S* rRNA gene of *C. difficile* (Cd198, 5′-Cy5-CATCCTGTACTGGCTCAC-3′; ref. 32) and EUB338 (universal all-bacterial *16S* probe, 5′-Cy3-GCTGCCTCCCGTAGGAGT-3′) for 2 hours at 46°C followed by DAPI counterstain as previously described (ref. 1; Supplementary Methods).

### Vero Cell Toxicity Assay

*C. difficile* toxin cytotoxicity assays were performed with Vero cells (RRID:CVCL_0059) as previously described (ref. 3; see Supplementary Methods).

### IHC

Paraffin-embedded, formalin- or Carnoy's-fixed mouse colonic sections were stained with anti-CD3 (catalog #99940, Cell Signaling, RRID:AB_2755035), anti–Ly-6G (catalog #87048, Cell Signaling, RRID:AB_2909808), or anti-F4/80 (catalog #70076, Cell Signaling, RRID:AB_2799771) antibodies (see Supplementary Methods). Immune cell infiltration was quantified using the HALO platform (Indica Labs).

### Immunofluorescence

Immunostaining was performed as previously described (33) with minor modifications (Supplementary Methods) using the primary antibodies anti–β-catenin (clone 12F7D1, Vanderbilt Antibody Protein Resource), anti–Ki-67 (clone 16A8, BioLegend, RRID:AB_11203533), and anti-PCNA (clone PC10, BioLegend, RRID:AB_314692) and secondary donkey anti-rat and goat anti-mouse antibodies (Thermo Fisher).

### High-Dimensional Flow Cytometry

GF *Apc^Min/+^* mouse colons were harvested and flushed with sterile PBS. The distal and mid colonic regions were collected, minced, and enzymatically digested as previously described (4); stained with a panel of intracellular and cell-surface antibodies (Supplementary Table S4); and analyzed on a FACSymphony flow cytometer (BD Bioscience; Supplementary Methods).

### 16S rRNA Amplicon Sequencing

16S rRNA amplicon sequencing of the V1V2 region was performed and analyzed as previously described (ref. 34; Supplementary Methods).

### Whole-Genome Sequencing of *C. difficile* Strains

DNA was extracted directly from microbial cultures of individual *C. difficile* strains and sequenced at the CHOP Microbiome Center, as described below. Microbial culture samples were shaken at 20,000 × *g* for 2 minutes, and then 20 μL of supernatant was taken from each sample tube and added to its corresponding well containing 30 μL of nuclease-free water in a 96-well PCR plate. Extraction cleanup was completed using SPRI/AmpureXP beads in a 50% PEG 8000 solution. DNA was then quantified using the Quant-iTTM PicoGreenTM dsDNA assay kit (Thermo Fisher Scientific) before library generation. Shotgun libraries were generated from 0.2 ng/μL DNA using the Illumina DNA Prep Kit (formerly Nextera FLEX Library Prep Kit) and IDT for Illumina unique dual indexes at 1:4 scale reaction volume. Library success was assessed by the Quant-iTTMPicoGreenTM dsDNA assay after AMPure cleanup of FLEX libraries using SPRI/AMPureXP beads in a 50% PEG 8000 solution. Samples with library yields <1 ng/μL were reprepped as needed. Library quality control was assessed through Fragment Analysis using the High-Sensitivity NGS Fragment Kit. All library samples were then pooled at an equal volume. Quality control of the library pool was performed on the Agilent BioAnalyzer to check the size distribution and absence of additional adaptor fragments. This quality control pool was then sequenced using a 300-cycle Nano kit on the Illumina MiSeq. Libraries were then repooled based on the demultiplexing statistics of the MiSeq Nano run. Final repooled libraries went through cBot clustering using HiSeq PE Cluster Kit v4 Box 1 of 2 prior to high-throughput sequencing using 250-cycle HiSeq SBS Kit v4 Boxes 1 of 2 and 2 of 2, as well as HiSeq PE Cluster Kit v4–cBot Box 2 of 2 on the Illumina HiSeq. Extraction blanks and nucleic acid–free water were processed along with experimental samples to empirically assess environmental and reagent contamination. A laboratory-generated mock community consisting of DNA from *Vibrio campbellii* and Lambda phage was included as a positive sequencing control.

Whole-genome alignment of sequenced and reference isolates was performed using MUGSY (35), followed by FastTree (36) for phylogenetic tree construction.

### scRNA-seq

Six GF *Apc^Min/+^* mice were gavaged with either the 3728T slurry (*n* = 3) or the 3979T slurry (*n* = 3) and housed in isocages for 2 weeks. At 2 weeks p.i., GF mice were shipped in GF shipping containers to the Vanderbilt University Medical Center for scRNA-seq. Mice were allowed to rest for 1 to 2 days prior to euthanasia. Colons were excised and flushed with cold sterile PBS. A small portion was reserved for histology. The remainder of each colon underwent chelation at 4°C, followed by mechanical and enzymatic dissociation using a cold-activated protease-DNase approach into single-cell suspensions, which were then encapsulated into droplets using the inDrop platform following the protocol of Banerjee and colleagues (37) and Adam and colleagues (38). Libraries were prepared using the TruDrop structure and sequenced using NovaSeq6000 (39). Raw reads were processed into counts matrices with an open-source pipeline (40, 41), and true cells were identified and filtered from barcodes using dropkick (42). Downstream analysis involving uniform manifold approximation and projection (UMAP) and heat map plots followed a standard framework (43) using Scanpy (44). Ranked gene lists from Scanpy-based differential gene expression analysis of 3728T slurry– versus 3979T slurry–gavaged mice at 2 weeks p.i. were used as input for the gene set enrichment analysis with WebGestalt (22). Cell type–specific ranked gene lists were chosen based on cluster analysis. The analyses [e.g., Kyoto Encyclopedia of Genes and Genomes (KEGG), Reactome] were chosen as functional database pathways in the basic parameters, and the preset defaults were used as advanced parameters. The most interesting of the significantly enriched gene sets were sorted by a normalized enrichment score (enrichment scores normalized to all permutations of the data sets). The size column refers to the number of genes in each set, and the leading-edge number is the number of ranked genes in the subset that contributes most to the enrichment score. The empirical false discovery rate is shown, because all *P* values were zero (*P* < 2.220446e−16 cannot be displayed by WebGestalt, so a zero is given in these circumstances). Downstream analysis was performed using the upstream regulator module of IPA (45), with differentially expressed genes with a *P* < 10e−5. Visual representation of modular networks was performed on upstream regulators designated as transcription factors as previously described (46).

### Gene Expression Assays (qRT-PCR)

RNA was extracted from mouse colons using standard TRIzol extractions as described in the Supplementary Methods and analyzed via TaqMan assays: *Nos2*, Mm00440502_m1; *Duox2*, Mm01326247_m1; *Il17A*, Mm00439618_m1; and Mouse *Gapdh* Endogenous Control (VIC/MGB probe, Primer Limited; Thermo Fisher Scientific).

### Statistical Analysis

Tumorigenesis, high-dimensional flow cytometry, and IHC statistics were performed in GraphPad Prism version 9.1. Graphs with multiple comparisons were first analyzed by Kruskal–Wallis; if significant (*P* < 0.05), subsequent Mann–Whitney *t* tests were performed.

### Data Availability

16S rRNA amplicon sequencing data are deposited in the NCBI Sequence Read Archive (SRA) under BioProject accession number PRJNA741397. scRNA-seq data are deposited in the Gene Expression Omnibus (GEO) at the NCBI under accession number GSE200969.

## Supplementary Material

Supplementary Figure

Supplementary Table

Supplementary Table

Supplementary Table

Supplementary Table
