# Supplementary material for: Human Colon Cancer–Derived Clostridioides difficile Strains Drive Colonic Tumorigenesis in Mice
Source: Cancer Discov. 2022 Jun 9;12(8):1873–85. doi: 10.1158/2159-8290.CD-21-1273 (PMC9357196; doi:10.1158/2159-8290.CD-21-1273)
Supplement: Supplementary Figure [file cd-21-1273_supplementary_figures_and_methods_suppsm1.docx]

Supplementary Materials and Methods

*C. difficile* spore preparation

For GF *Apc^Min/+^* mouse experiments, *C. difficile* spores were created using Clospore media (1). Briefly, 2 mL of an actively growing BHI broth culture of *C. difficile* were sub-cultured into a 40 mL aliquot of Clospore media in the anaerobic chamber at 37°C for 6-7 days. Spores were then washed 3-5x with 40 mL of cold sterile water, centrifuged at 3200 rpm for 20 min at 4°C. Spore stocks were resuspended in 1 mL sterile water in a screw-cap tube and stored at 4°C. Spore stocks were heat-inactivated at 65°C for 20 min and plated in 10-fold dilution series onto TCCFA plates to enumerate spores. 10^4^ spores of the Cd CIm_3728T or CIm_3752T strains were gavaged into each mouse in combination with the 3728T isolates. For the pathogenic M7404 strain, a range of 10^2^ - 10^4^ spores were gavaged due to high lethality with 10^4^ spores with the TcdB^+^ strains.

For SPF *Apc^Min/+^* mouse experiments, *C. difficile* strains, ATCC 9689, ATCC 700057, and CRC-associated isolate CI_m__2663_BF^+^T were plated as a lawn on reduced BHI plates and cultured anaerobically for 5 to 8 days at 37°C to induce sporulation. Colonies were collected in 1-2 mL sterile PBS and incubated at 60°C for 1 hour to kill the vegetative cells. Spores were washed 3 times with PBS, and viable spores were enumerated as colony-forming units (CFUs)/mL by plating serial dilutions on CCFA plates with horse blood and taurocholate (CCFA-HT) plates or BHI agar supplemented with 0.1% taurocholate (BHI-T).

Vero cell toxicity assay

*C. difficile* toxin cytotoxicity assays were performed as previously described (3). Briefly, green African monkey kidney epithelial (Vero, ATCC CCL-81, RRID:CVCL_0059 passage 3-30) cells were grown to confluence in Dulbecco modified Eagle medium (DMEM, Gibco Laboratories) with 1% penicillin-streptomycin (Gibco Laboratories) and 10% fetal bovine serum (Gibco Laboratories) prior to plating at a density of 10^5^ cells per well in a 96-well plate. Mycoplasma testing was not routinely performed. Fecal samples were diluted 1/10 w/v and homogenized in sterile PBS. Fecal debris was pelleted by centrifugation at 13,000 x *g* for 5 min. Ten-fold serial dilutions of supernatants were added to Vero cell wells and incubated overnight at 37°C with 10% CO_2_ in duplicate or triplicate. Cell rounding cytotoxicity titers were calculated as the log_10_ of the reciprocal value of the highest dilution with 50-100% rounding of cells in a given well normalized to starting stool weights.

Quantitative real-time PCR (qPCR) for *C. difficile 16S* rRNA gene and toxin status

For determination of toxin status of *C. difficile* strains, single colonies were picked from BHI plates and boiled in 50 µL of sterile water, centrifuged 5 min at 10,000 x *g*, and supernatants reserved for qPCR. Five µL of boiled lysate supernatants were combined with 0.4 µM forward and reverse primers for either TcdA or TcdB genes and SYBRGreen Master Mix in a final volume of 25 µL. Samples were run on an Applied Biosystems qPCR machine with the following cycles: 50°C for 2 min, 95°C for 10 min, 40 cycles of 95°C for 30 sec and 58°C for 1 min. Primers were as follows: TcdA F: 5’-GTCGGATTGCAAGTAATTGACAATA-3’, TcdA R: 5’-TAACAGTCTGCCAACCTTTTGAGA-3’, TcdB F: 5’-ACCATATAGCTTTGTAGATAGTGAAGGAAA-3’, TcdB R: 5’-AAGAACTACATCAGGTAATTCAGATACAAA-3’ (2).

Gene expression in mouse colons

For determination of host gene expression in mouse tissues, RNA was extracted from biopsies of mouse colonic tissues using 1.0 mm diameter Zirconia beads (BioSpec), homogenization with TRIZOL, and bead beating for 2 x 1 min in a Mini-Beadbeater-96 cell disrupter (Biospec Products) followed by standard phenol/chloroform extraction. Samples were DNase1-treated (Sigma) before undergoing reverse transcription with the High-Capacity RNA-to-cDNA Kit (Applied Biosystems). cDNA was subsequently analyzed for gene amplification with the TaqMan 2X Gene Expression Mix (Thermo Fisher Scientific) for the following pre-validated TaqMan FAM assays: *Nos2*, Mm00440502_m1; *Duox2*, Mm01326247_m1; and *Il17A*, Mm00439618_m1. Mouse *Gapdh* Endogenous Control (VIC/MGB probe, primer limited) (Thermo Fisher Scientific) was used as a housekeeping control. Data were analyzed by the delta delta Ct method.

Fluorescence in situ hybridization (FISH)

Paraffin-embedded, Carnoy’s- (60/30/10 methanol/acetic acid/chloroform) or poloxamer- (20% poloxamer, 80% cold neutral-buffered formalin w/v) (3) fixed unflushed mouse colons or human CRC specimens were stained with a probe targeting the *16S* gene of *C. difficile* (Cd198, 5’-Cy5-CATCCTGTACTGGCTCAC-3’) (4) and EUB338 (universal all-bacterial *16S* probe, 5’-Cy3-GCTGCCTCCCGTAGGAGT-3’) for 2 h at 46°C followed by DAPI counterstain as previously described (5). Slides were imaged on a Zeiss 780 laser-scanning confocal microscope. Samples were designated as biofilm positive (BF^+^) if at least one region contained mucus-invasive bacterial aggregates (>20 bacteria) that spanned 200 µm of the epithelial tissue and were within 1 µm of the epithelial surface.

To quantitate *C. difficile* tissue invasion in the M7404 2 wk p.i. experiments, poloxamer-fixed, paraffin-embedded slides were stained with Cd198 in Cy3 and EUB338 in Cy5. Five images at 40X were acquired from each mouse distal colon in a blinded, randomized manner, choosing regions that had intact mucus and good crypt orientation. The number of tissue-invasive *C. difficile* bacteria observed invading into the tissue (not just into the crypts) were then averaged across the 5 images for each sample.

Immunohistochemistry (IHC)

Mouse colons were fixed in 10% formalin or Carnoy’s solution followed by paraffin-embedding and sectioning. Unstained colonic sections were subjected to IHC staining for immune cells using the following antibodies: anti-CD3 (Catalog #99940, Cell Signaling, USA, RRID:AB_2755035), anti-Ly-6G (Catalog #87048, Cell Signaling, USA, RRID:AB_2909808), and anti-F4/80 (Catalog #70076, Cell Signaling, USA, RRID:AB_2799771). In brief, the slides were dewaxed with xylene, rehydrated through an ethanol gradient, underwent antigen retrieval with citrate buffer (pH 6), followed by treatment with 3% hydrogen peroxide. Immediately after the saturation of non-target binding sites, the primary antibodies were applied onto tissue sections for an overnight incubation at 4°C. The next day, the slides were washed with TBST buffer (Tris-Buffered Saline 0.1% TWEEN ®20, Sigma) and incubated with a secondary antibody (anti-rabbit IgG, Abcam) for 30 min at room temperature. The slides were developed with DAB (3,3’-Diaminobenzidine, Abcam) reagent, followed by TBST washes, hematoxylin counterstain, dehydration through an ethanol gradient and mounting with coverslips. Immune cell infiltration of normal regions in distal colons were quantified using the HALO platform (Indica Labs) in the Tumor Microenvironment Lab at the Johns Hopkins University School of Medicine.

Immunofluorescence (IF)

Immunofluorescent staining on mouse colonic tissues was performed as previously described (6). Briefly, paraffin-embedded, poloxamer-fixed colonic tissue slides were deparaffinized with Histoclear (National Diagnostics) and permeabilized with Phosphate Buffered Saline (PBS) including 0.3% Triton X-100. Tissue was blocked with PBS containing 10% Normal Donkey Serum (NDS) and 3% Bovine Serum Albumin (BSA). Primary antibodies were diluted in PBS containing 5% NDS and 1.5% BSA. Tissue was incubated with primary antibody overnight at 4°C. Tissue was washed with PBS three times before incubating with secondary antibodies (in PBS + 5% NDS + 1.5% BSA) and Hoechst 33342 (20 μM) nuclear stain at room temperature for 1 h. Tissue sections were washed again with PBS and coverslips were mounted with Fluoromount-G (Southern Biotech). Primary antibodies used were anti-β-catenin (clone 12F7D1, Vanderbilt Antibody Protein Resource), anti-Ki67 (clone 16A8, BioLegend, RRID:AB_11203533), and anti-Peripheral Cell Nuclear Antigen (anti-PCNA, clone PC10, BioLegend, RRID:AB_314692) conjugated to Cy5. Secondary antibodies were donkey anti-rat AlexaFluor 568 and goat anti-mouse AlexaFluor 488 (both from ThermoFisher). Images were acquired on a Leica DM6000 B microscope at 20x magnification.

High-dimensional flow cytometry

Six GF *Apc^Min/+^* mice were gavaged with the 3728T isolates +/- 10^4^ spores of the *C. difficile* CIm_3728T strain (*N* = 5-6 per group). After 2 wk, colons were harvested and flushed with sterile PBS. The distal and mid colon regions were collected, minced, and enzymatically digested as previously described (7). Cells were stained using the antibodies described in **Table S4**. Intracellular cytokine staining was performed following 3.5 h stimulation in Iscove’s Modified Dulbecco’s Medium (IMDM) with 5% fetal calf serum (FCS) in the presence of Stimulation Cocktail (Ebioscience), which contains phorbol 12-myristate 13-acetate (PMA) and ionomycin. After a PBS 1X wash, cells were stained with cell surface antibodies (**Table S4**) for 30 min on ice. After a PBS 1X wash, cells were next incubated (20 min, room temperature) with FoxP3 fixation/permeabilization buffer (Ebioscience) followed by a single wash with PermWash buffer 1X (BD Bioscience) and then stained 30 min on ice with anti-Foxp3 and cytokine antibodies diluted in PermWash buffer 1X. After a final wash with Perm/Wash buffer 1X, samples were run on the FACSymphony flow cytometer (BD Bioscience). All sample data was exported as FCS files and each was gated on live singlet. Optimized t-Distributed Stochastic Neighbor Embedding (Opt-SNE) was performed for dimension reduction analysis, phenograph and FlowSOM for clustering analysis on a panel of up to 32 markers. Opt-SNE was run at perplexity of 30. FlowSOM was set to render 23 metaclusters for myeloid and ILC embedding whereas FlowSOM clustering was set for 25 metaclusters with the same Opt-SNE hyperparameters in the T and B cell visualizations.

*16S* rRNA gene amplicon sequencing

DNA from mouse stool, human CRC tissue slurries, or the 3728T isolate mixture were extracted using the Zymo Research 96-well Quick-DNA Fecal/Soil Microbe Kit according to the manufacturer’s instructions, including bead bashing in 2 mL tubes with 0.1 and 0.5 mm beads in buffer with a Mini-Beadbeater-96 cell disrupter (Biospec Products). Extraction blanks and DNA-free water were subjected to the same amplification and purification procedure to allow for empirical assessment of environmental and reagent contamination. Positive controls consisting of eight artificial 16S gene fragments synthesized in gene blocks and combined in known abundances were also included. Each 96-well plate also included two additional standard controls for comparison across MiSeq runs: Zymo Microbial community standard catalog (#D6300) and Zymo Microbial Community Standard II Log Distribution (#D6310). Samples were then shipped to Children’s Hospital of Pennsylvania (CHOP) Microbiome Center for amplification and sequencing as follows: barcoded PCR primers annealing to the V1-V2 region of the *16S* rRNA gene were used for library generation (V1-V2 Forward primer: 5'-AGAGTTTGATCCTGGCTCAG-3’; V1-V2 Reverse primer: 5'-TGCTGCCTCCCGTAGGAGT-3'). PCR was carried out in quadruplicate using Q5 High-Fidelity DNA Polymerase (NEB, Ipswich, MA). Each PCR reaction contained 0.5 µM of each primer, 0.34 U Q5 Pol, 1X buffer, 0.2 mM dNTPs, and 2.5 µL (high biomass samples) or 5.0 µL (low biomass samples) DNA in a total volume of 25 µL. Cycling conditions were as follows: 1 cycle of 98°C for 1 m; 20 or 25 cycles of 98°C for 10 s, 56° C for 20 s, and 72°C for 20 s; 1 cycle of 72°C for 8 m. After amplification, quadruplicate PCR reactions were pooled and then purified using a 1:1 volume of Agencourt AmPure XP beads (Beckman-Coulter, Brea, CA, USA). DNA in each sample was then quantified using PicoGreen and pooled in equal molar amounts. The resulting library was sequenced on the Illumina MiSeq or HiSeq using 2x250 bp chemistry.

Taxonomic assignment of *16S* rRNA amplicon sequence data

Paired-end Illumina *16S* rRNA amplicon sequences (V1V2 region) were pre-processed for quality using Trimmomatic (v.0.32) (min length 200 bp; min Phred quality score 20 over a 25 bp sliding window) (8) with read merging using FLASH (v.1.2.7) (min. overlap 20 bp; 5% max. mismatch density) (9). Passing merged sequences were evaluated for quality and length using QIIME (v.1.8.0) (min. final length 200 bp) (10). Sequences matching spike-in PhiX-174 controls were filtered using BLASTN (v.2.2.22), followed by chimera detection with UCLUST (v.1.2.22q) (11), and removal of human-associated contaminant using Bowtie2 (v.2.2.4) (12). Reads with assignments to chloroplast or mitochondrial contaminants by the RDP classifier (v.2.2) (13) (min. confidence 50%) were also filtered. The final set of passing*16S* rRNA amplicon sequences were assigned to high-resolution taxonomic membership by Resphera Insight (v.2.2) (Baltimore, MD) (5,14,15).

*C. difficile* ribotyping

DNA from single colonies of *C. difficile* strains were extracted using the Zymo Research Quick-DNA Fecal/Soil Microbe Kit according to the manufacturer’s instructions, including bead bashing with a Fast-Prep 24 homogenizer (MP Biomedicals). Fluorescent PCR ribotyping was performed as previously described at Montana State University (16). Briefly, fluorescent amplicons spanning intergenic regions between the 16S and 23S rRNA encoding genes were generated by PCR using a standard forward primer (GTGCGGCTGGATCACCTCCT) and a 6-carboxyfluorescein (FAM)-labeled reverse primer (56-FAM/CCCTGCACCCTTAATAACTTGACC). Fragments were then sized using an ABI3730XL capillary electrophoresis machine (i.e. Sanger sequencer) and compared to a database containing >20,000 ribotyped isolates from clinical cases of disease (thewalklab.com/tools). The analysis pipeline currently identifies 125 distinct ribotypes, 35 of which are cognate with the most prevalent pathogens in the UK *C. difficile* ribotyping network (CDRN) based on whole genome sequence comparisons. FP485 was identified previously in clinical samples from a multiyear, multicenter surveillance study in the US, where it comprised ~2% of all isolates evaluated (17).


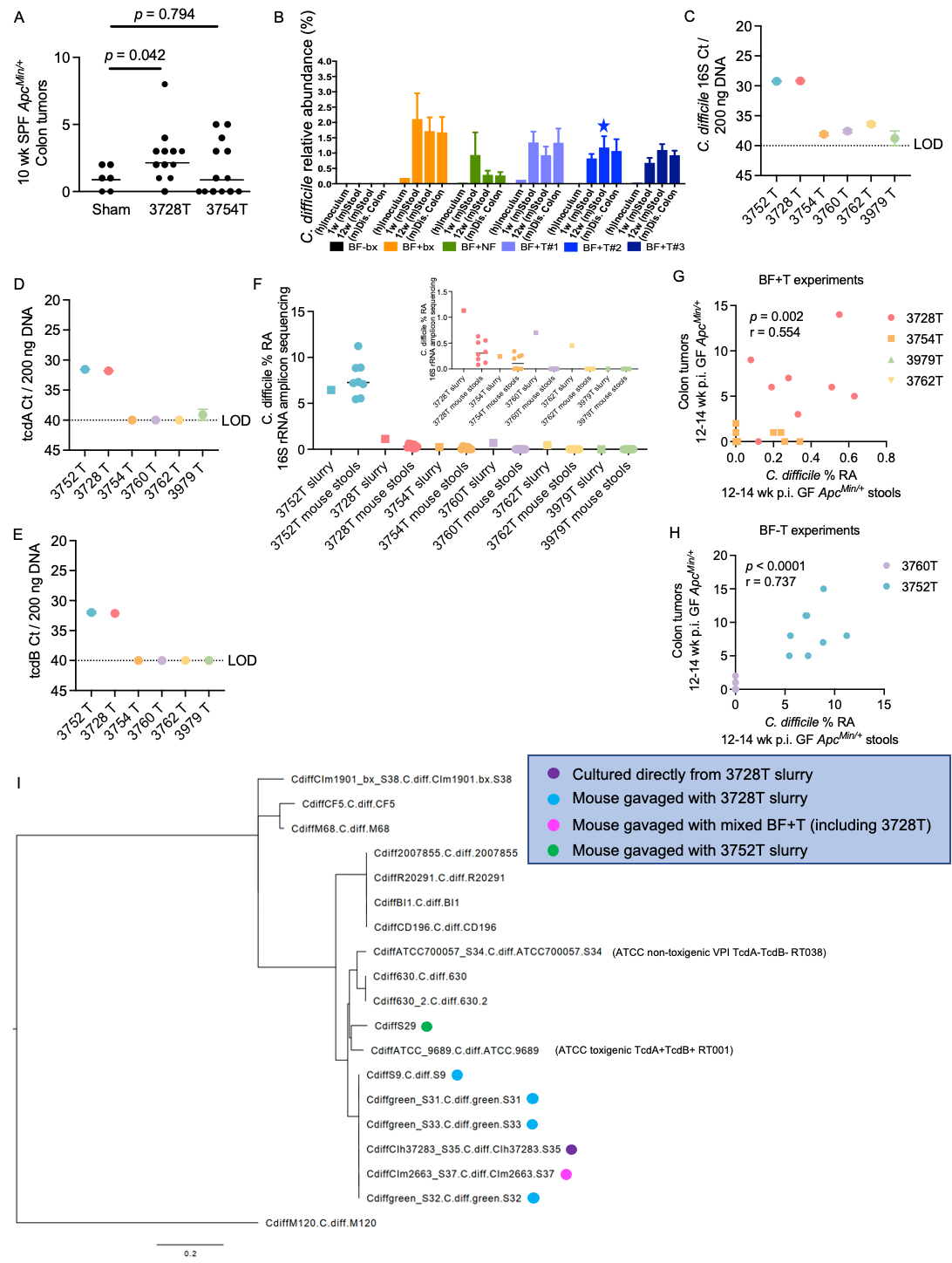


Fig. S1. The human CRC-derived 3728T slurry and *C. difficile* are tumorigenic in *Apc^Min/+^* mice. (A) Tumorigenicity of the 3728T slurry in SPF *Apc^Min/+^* mice. Three days prior to inoculation, SPF *Apc^Min/+^* were gavaged with 100 μL of cefoxitin (500 μg/mL) and given cefoxitin (500 mg/L) in their drinking water for 2 days. Following a 1 day wash out period, mice were gavaged with 100 μL of either the 3728T slurry or 3754T slurry, two components of the original mixed biofilm-positive tumor slurry (BF^+^T) that was tumorigenic in prior mouse studies (18). Mouse colons were harvested at 10 wk p.i. and examined for colonic tumors. Mann-Whitney *p*-values are shown. (B) *C. difficile* relative abundance in murine tumorigenesis studies performed with mixed human mucosal inocula. Mixed human mucosal inocula were made by combining colonic mucosa from biofilm-negative biopsies (BF^-^bx) obtained during screening colonoscopies of 5 healthy individuals or biofilm-positive colon tumors (BF^+^T) or paired normal flanking tissues (BF^+^NF) obtained during colonic resections from 5 CRC patients. These inocula were gavaged into GF *Apc^Min∆850/+^* and *Apc^Min∆850/+^*;*Il10^-/-^* mice as previously described (18). BF^+^T#1, BF^+^T#2, BF^+^T#3 were inocula made separately at different times from the same 5-patient CRC mixture and included the 3728T and 3754T slurries. The blue star denotes the mouse from which the *C. difficile* strain CIm_2663_BF^+^T was cultured. (C-E) qRT-PCR of individual patient BF^+^ and BF^-^ CRC tumor slurries for the *C. difficile 16S*, *tcdA*, and *tcdB* genes. Dashed line represents the limit of detection of the assay (LOD). The vertical axis is inverted to aid in interpretation of the Ct values. Two hundred ng of DNA were tested for all samples except for 3754T, for which only 20 ng of DNA was utilized due to limited availability of that specimen. (F-H) 16S rRNA amplicon sequencing of individual patient BF^+^ and BF^-^ CRC tumor slurries and 14 wk p.i. stools from GF *Apc^Min/+^* mice gavaged with the 3728T slurry. (F) *C. difficile* relative abundance in the patient slurries and corresponding mouse stools. Inset magnifies the lower abundance samples. Mouse stool *C. difficile* relative abundance vs. mouse colonic tumors in mice gavaged with (G) BF^+^ or (H) BF^-^ slurries. In G, the 3979T and 3762T mouse samples all had 0 *C. difficile* reads, 0-1 tumors, and are plotted at the juncture of the x/y axis but are not visible. (I) Phylogenetic tree of *C. difficile* strains. *C. difficile* strains isolated from the stools or distal colons of mice gavaged with the mixed BF^+^T slurry, 3728T slurry, or 3752T slurry or isolated directly from the 3728T slurry underwent whole genome sequencing. Phylogenetic analyses demonstrated that the 3728T-associated strains (purple and blue circles) shared a high degree of similarity (>99%) with the CIm_2663 isolate cultured from the mixed BF^+^T slurry-gavaged mice (pink), indicating that these strains arose from a common ancestor. Further analyses indicated the 3728T-derived *C. difficile* strains were of ribotype (RT) FP485. In contrast, the isolate derived from mice gavaged with the 3752T slurry (green) belonged to a divergent group more closely related to the ATCC toxigenic 9689 strain and was subsequently determined to be of RT 014-020. RT 014-020 is a leading *C. difficile* RT in the United States (19). Other *C. difficile* strains included in the phylogenetic tree are 10 reference strains obtained from NCBI (CF5, M68, 2007855, R20291, BI1, CD196, 630, 630.2, and ATCC 9689), as well as the ATCC 700057 strain, which was sequenced as part of the present study.


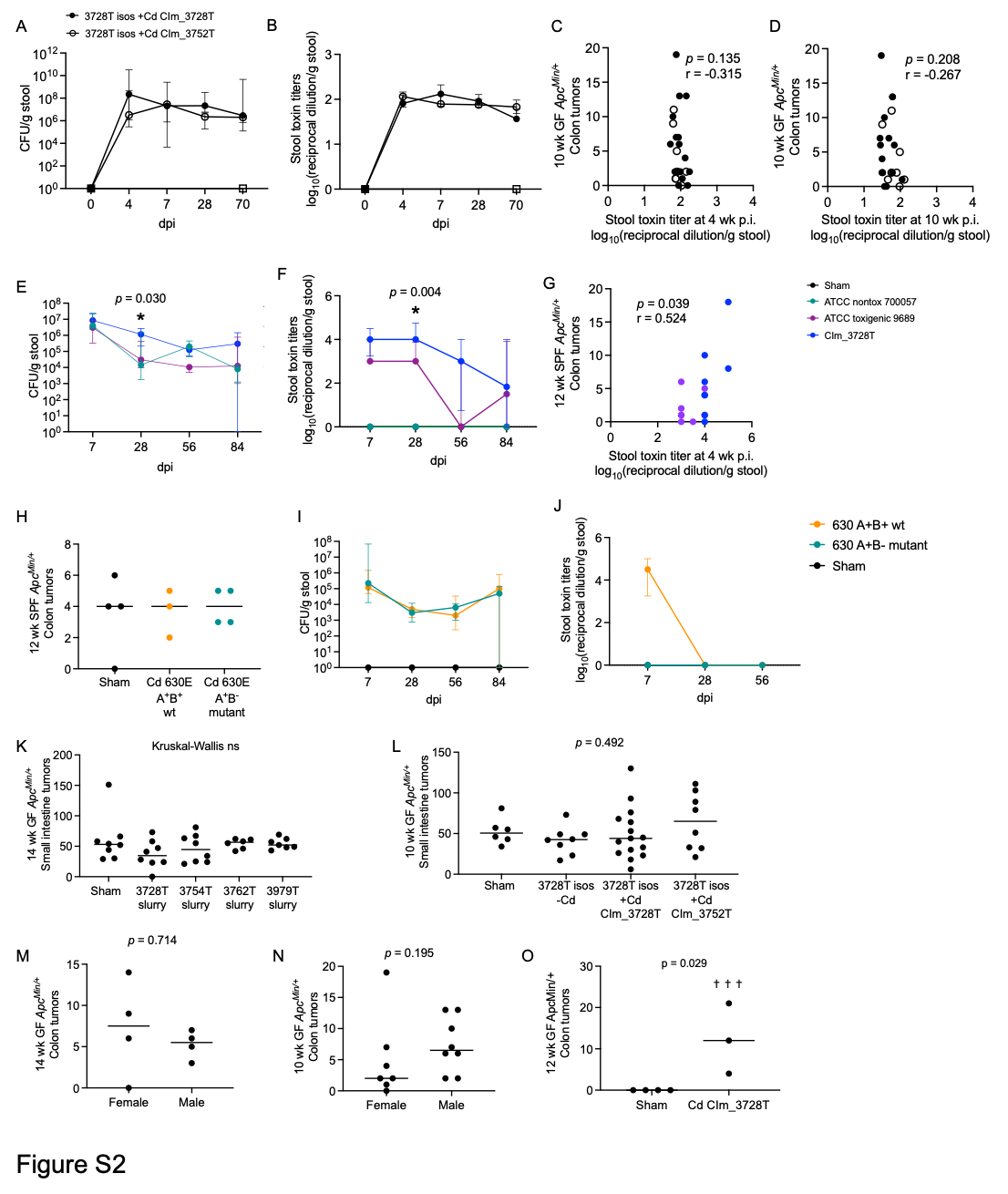


**Fig. S2. *C. difficile* colonic tumorigenesis is associated with chronic toxin production by virulent strains.** (**A**) Longitudinal stool *C. difficile* colony forming units (CFUs) and (**B**) stool toxin titers (measured by Vero cell rounding assay, which indiscriminately measures both TcdA and TcdB) in GF *Apc^Min/+^* mice gavaged with the 3728T isolates + *C. difficile* strains CIm_3728T or CIm_3752T over the course of a 10-wk experiment. (**C,D**) Spearman correlation of toxin titers at 4 wk p.i. or 10 wk p.i. vs. colonic tumors at 10 wk p.i. (**E**) Stool CFUs and (**F**) toxin titers in a vancomycin/gentamicin chronic SPF *Apc^Min/+^* model with toxigenic *C. difficile* strains. A non-toxigenic strain (ATCC 700057) also chronically colonized these mice, but as expected, did not produce detectable levels of toxin. *p*-values in E and F represent Mann-Whitney analyses of stool toxin titers at day 28 p.i. between CIm_3728T and ATCC toxigenic 9689-gavaged mice. (**G**) Stool toxin titers at 4 wk p.i. correlated with tumor counts at 12 wk in the SPF *Apc^Min/+^* model. (**H-J**) The 630Δerm strain does not induce tumors in SPF *Apc^Min/+^* mice. Using the vancomycin/gentamicin chronic SPF *Apc^Min/+^* colonization model, mice were gavaged with wild-type (WT) or TcdB^-^ mutant strains of the 630Δerm *C. difficile* strain. (**H**) Colonic tumor counts revealed that this strain did not significantly induce tumorigenesis above sham controls despite successful chronic colonization, as determined by CFU/g stool (**I**). (**J**) Toxin was only detected acutely in the mice at day 7 p.i. (**K,L**) *C. difficile* does not alter small intestinal tumorigenesis. Small intestines from GF *Apc^Min/+^* mice gavaged with either the (**K**) 3728T slurry or (**L**) 3728T isolates with various *C. difficile* strains demonstrated that there was no difference in tumorigenesis in the small intestine at 10-14 wk p.i. compared to sham controls. The *p*-values represent Kruskal-Wallis analyses in **K** and **L**. (**M,N**) No sex differences in *C. difficile*-associated tumorigenesis in mice. Colons from GF *Apc^Min/+^* mice gavaged with either the (**M**) 3728T slurry or (**N**) 3728T isolates with the CIm_3728T *C. difficile* strain demonstrated that there was no sex difference in tumorigenesis in the colons at 10-14 wk p.i. Mann-Whitney *p*-values are shown. (**O**) Colonic tumor counts from GF *Apc^Min/+^* mice gavaged with spores from Cd CIm_3728T strains. Crosses represent mouse deaths. Mann-Whitney *p*-value is shown.


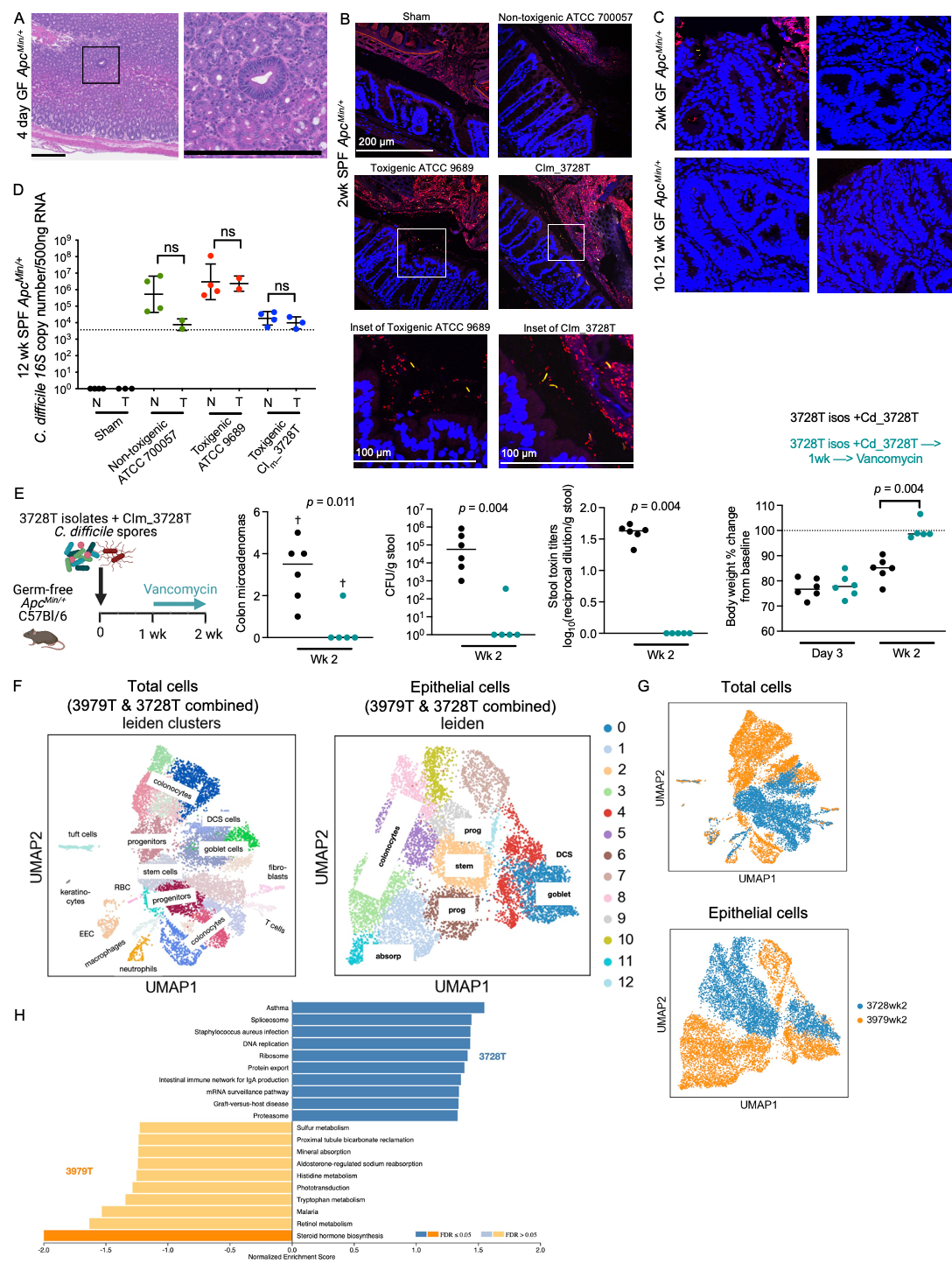


**Fig. S3. Early pro-tumorigenic events induced by the 3728T slurry and isolates and blockade with vancomycin.** (**A**) The *C. difficile*-containing 3728T slurry induces early dysplasia. Representative H&E staining of a distal colon with a dysplastic crypt (inset) from a GF *Apc^Min/+^* mouse at day 4 p.i. following gavage with the 3728T slurry. Scale bars: 200 μm. (**B**) Representative images of lack of mucus-invasive biofilms in the distal colons of SPF *Apc^Min/+^* mice. Colons were stained with the EUB338 and Cd198 probes in Cy3 and Cy5, respectively. (**C**) Representative FISH images at 2 wk and 10-12 wk p.i. in GF *Apc^Min/+^* mice demonstrating minimal to no *C. difficile* invasion of microadenomas and adenomas, respectively. (**D**) Distal colon tumor (T) vs. normal (N) tissue levels of *C. difficile* 16S RNA as measured by RT-PCR. (**E**) Diagram of vancomcyin experiment. GF *Apc^Min/+^* mice were gavaged with the 3728T isolates + CIm_3728T, then after 1 wk given vancomycin in their drinking water. Colons were harvested at 2 wk p.i. for microadenomas. Stool CFUs, toxin titers, and % change in body weight are also shown. *p*-values represent Mann-Whitney t-tests. (**F-H**) Single-cell RNA sequencing (scRNAseq) analysis of GF *Apc^Min/+^* mice gavaged with either the pro-tumorigenic 3728T slurry or non-tumorigenic 3979T slurry. Colons were harvested at 2 wk p.i. (**F**) UMAP plots of single-cell RNA sequencing (scRNAseq). Projections of leiden clusters for all cell types (**left**) or epithelial cells (**right**) identified by scRNAseq. **(G)** Divergent projections of single cells within the 3728T (blue) vs. 3979T (orange) slurry-gavaged mice for both total cell and epithelial cell analyses. (**H**) Gene set enrichment analysis of the 3728T vs. 3979T slurry-gavaged mice. Abbreviations: *DCS, deep crypt secretory cells; EEC, enteroendocrine cells; RBC, red blood cells; prog, progenitor cells; absorp, absorptive colonocytes.*


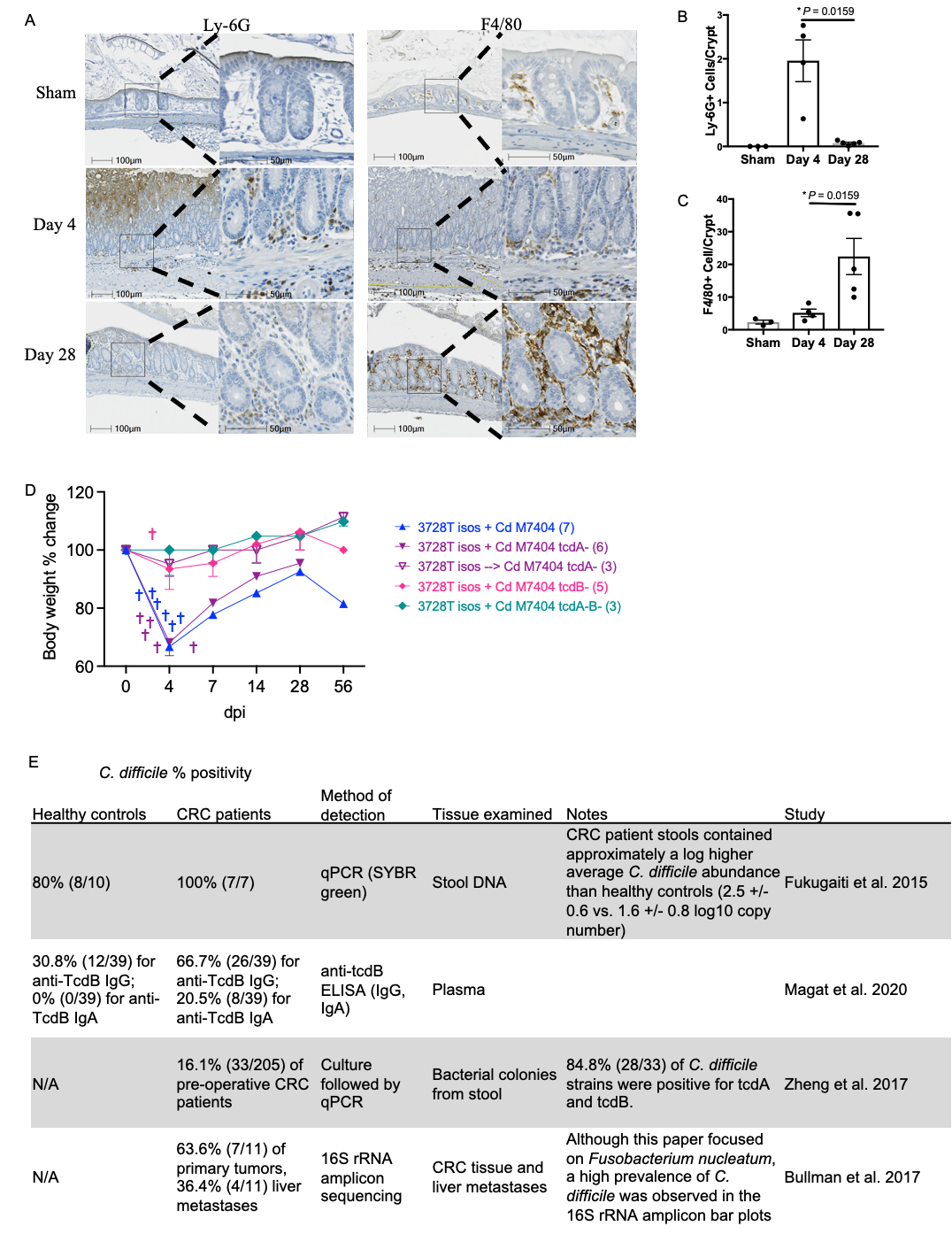


**Fig. S4.** (**A-C**) Immunostaining of Ly-6G^+^ and F4/80^+^ myeloid cells in GF *Apc^Min/+^* mice with the 3728T slurry. (**A**) Representative images of Carnoy’s-fixed distal colons from sham, day 4, or day 28 p.i. GF *Apc^Min/+^* mice were gavaged with the 3728T slurry and stained for (**left**) Ly-6G or (**right**) F4/80. Left panel images were obtained at 200x magnification, scale bars: 100 μm. Inset images were obtained at 400x magnification, scale bars: 50 μm. Positively stained cells in the lamina propria were quantitated using HALO software and normalized to the number of crypts within that region, spanning 100-200 crypts, for (**B**) Ly-6G and (**C**) F4/80. Mann-Whitney *p*-values are shown. (**D**) Longitudinal percent change in body weight for GF *Apc^Min/+^* mice gavaged with the 3728T isolates and either concomitant (+) or 1 wk delayed (🡪) gavage with spores from M7404 strains. (**E**) Table of prior studies that have reported positivity rates of *C. difficile* in CRC patients.


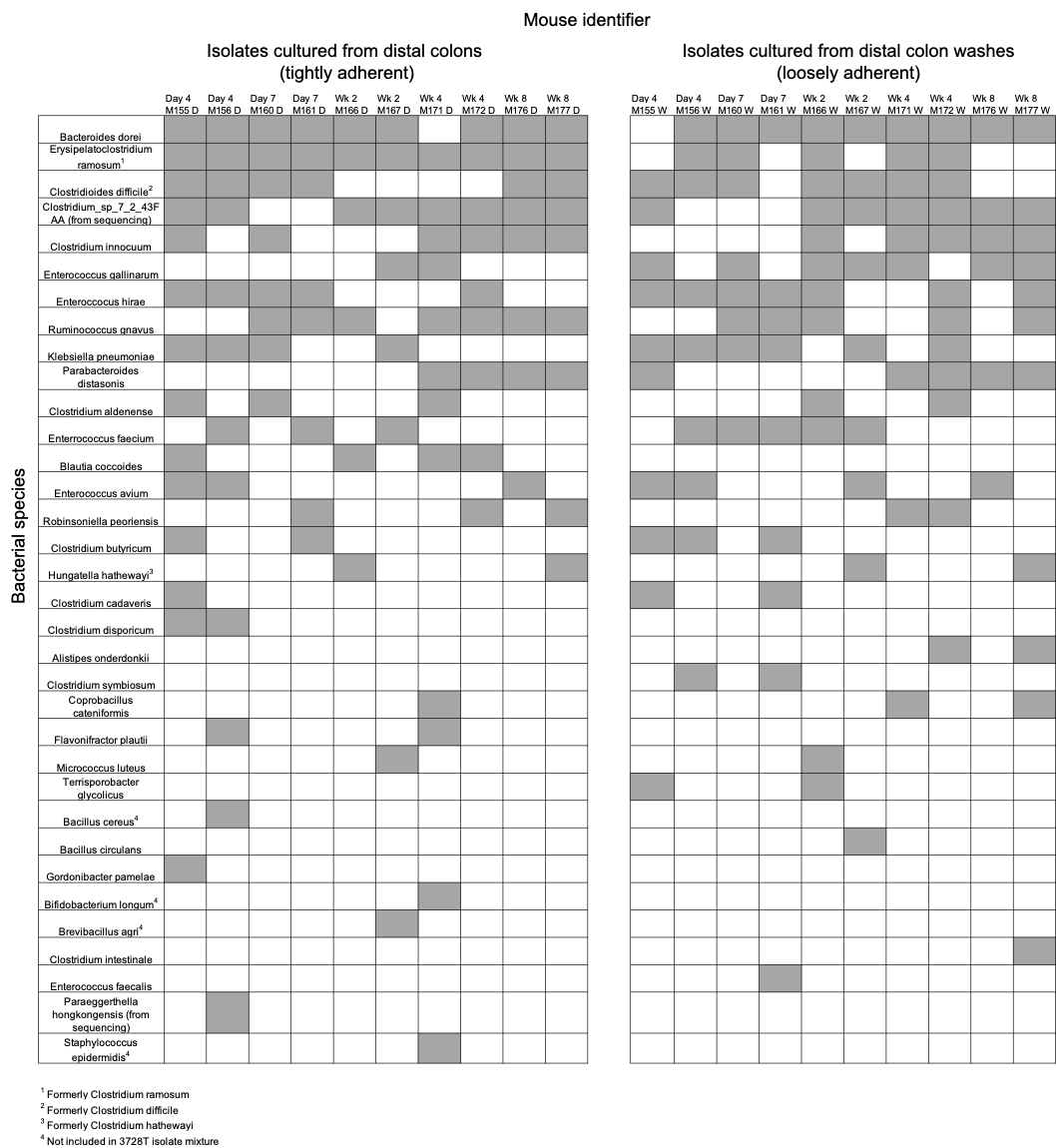


Table S1. Generation of the 3728T isolate mixture: culturing of colons from 3728T slurry-gavaged GF *Apc^Min/+^* mice. GF *Apc^Min/+^* mice were gavaged with the 3728T slurry and sacrificed at various time points p.i. (day 4, 7, 14, 28, 56). A 0.5 cm distal colonic biopsy from 2 mice per time point was washed 5x with sterile PBS and then homogenized. Both the residual tightly adherent bacteria from the homogenized biopsy and loosely adherent bacteria from the distal colon washes were extensively cultured on anaerobic and aerobic plates and broth to identify potential species associated with invasive biofilm formation vs. those associated with outer mucus bacterial communities. Cultivated isolates were identified by MALDI-TOF MS and/or 16S rRNA amplicon sequencing of individual colonies. Species are displayed in rank order from most frequently isolated (top) to least frequently isolated (bottom) in tightly and loosely adherent mouse samples. Successful cultivation and identification of a given bacterial isolate from a mouse is indicated with a gray shaded box. No major differences were observed between the tightly adherent (left panel) and loosely adherent (right panel) communities by culture. Three bacterial species were detected in all mice from either the tightly or loosely adherent communities: *Bacteroides dorei*, *Erysipelatoclostridium ramosum*, and *C. difficile*. To generate the 3728T isolate mixture, a single colony of each species was randomly chosen from the harvested isolates, cultured separately, and then combined into a final inoculum and stored in aliquots (see Materials and Methods). Four of the original bacterial isolates are denoted as not included in the final 3728T isolate mixture because they were either not able to be re-cultivated from frozen stocks or, in the case of *Staphylococcus epidermidis*, were presumed to be contamination. In addition to this list of microbes cultured from mice gavaged with the 3728T slurry, several culturing attempts were also performed directly on the 3728T slurry. Despite repeated attempts, no *E. coli* or *B. fragilis* strains (either toxigenic or non-toxigenic) were obtained. However, consistent with prior FISH analyses of this subject suggesting a prominent percentage of *Fusobacterium* spp. in the original patient’s CRC biofilm, a *F. nucleatum* strain was able to be cultivated directly from the 3728T slurry but was not able to successfully re-colonize the mice, and therefore was not included in the 3728T isolate mixture.

| *C. difficile* strain (CIm = Clinical Isolate from germ-free mouse) | TcdA status | TcdB status | Toxin status by Vero cell assay (detects both TcdA and TcdB) | Binary toxin status (cdtA / cdtB) | Ribotype | Source (CRC = colorectal cancer, BF = biofilm, T = tumor, GF = germ-free, CDI = *C. difficile* infection) | Notes on source | Reference |
| --- | --- | --- | --- | --- | --- | --- | --- | --- |
| CIm161_DC1_3728T (CIm_3728T) | + | + | + | -/- | 485 | CRC patient BF+T -> GF mouse | 3728T slurry-gavaged GF Min mice (distal colon tissue from mouse 161, distal colon isolate 1) | This study |
| CIm2663_BF+T mix (CIm_3728T) | + | + | + | -/- | 485 | CRC patient BF+T mix -> GF mouse | BF+T mixed slurry-gavaged GF Min mice (stool from mouse 2663 at University of Florida) | This study; Tomkovich and Dejea et al. 2019 |
| CIm313_3752T | + | + | + | -/- | 014-020 | CRC patient BF+T -> GF mouse | 3752T slurry-gavaged GF Min mice (stool from mouse 313) | This study |
| ATCC 700057 (VPI 11186) | - | - | nd | -/- | 038 |  | ATCC |  |
| ATCC 9689 (90556-M6S) | + | + | nd | -/- | 001 |  | ATCC |  |
| 630∆erm WT | + | + | nd | -/- | 012 | CDI patient | Parent 630 strain was isolated from a patient with pseudomembranous colitis and was associated with a hospital outbreak in Switzerland in 1979-1980; current strain has been extensively lab passaged | [Wust et al. 1982; Lyras et al. 2009](https://www.ncbi.nlm.nih.gov/pubmed/19252482) |
| 630∆erm tcdB- | + | - | nd | -/- | 012 | CDI patient |  | [Lyras et al. 2009](https://www.ncbi.nlm.nih.gov/pubmed/19252482) |
| M7404 WT (BI/NAP1/027) | + | + | + | +/+ | 027 | CDI patient | Canadian epidemic strain | Carter et al. 2015 |
| M7404 tcdA- | - | + | + | +/+ | 027 | CDI patient |  | Carter et al. 2015 |
| M7404 tcdB- | + | - | + | +/+ | 027 | CDI patient |  | Carter et al. 2015 |
| M7404 tcdA-B- | - | - | - | +/+ | 027 | CDI patient |  | Carter et al. 2015 |

Table S2. *C. difficile* strains used in this study.


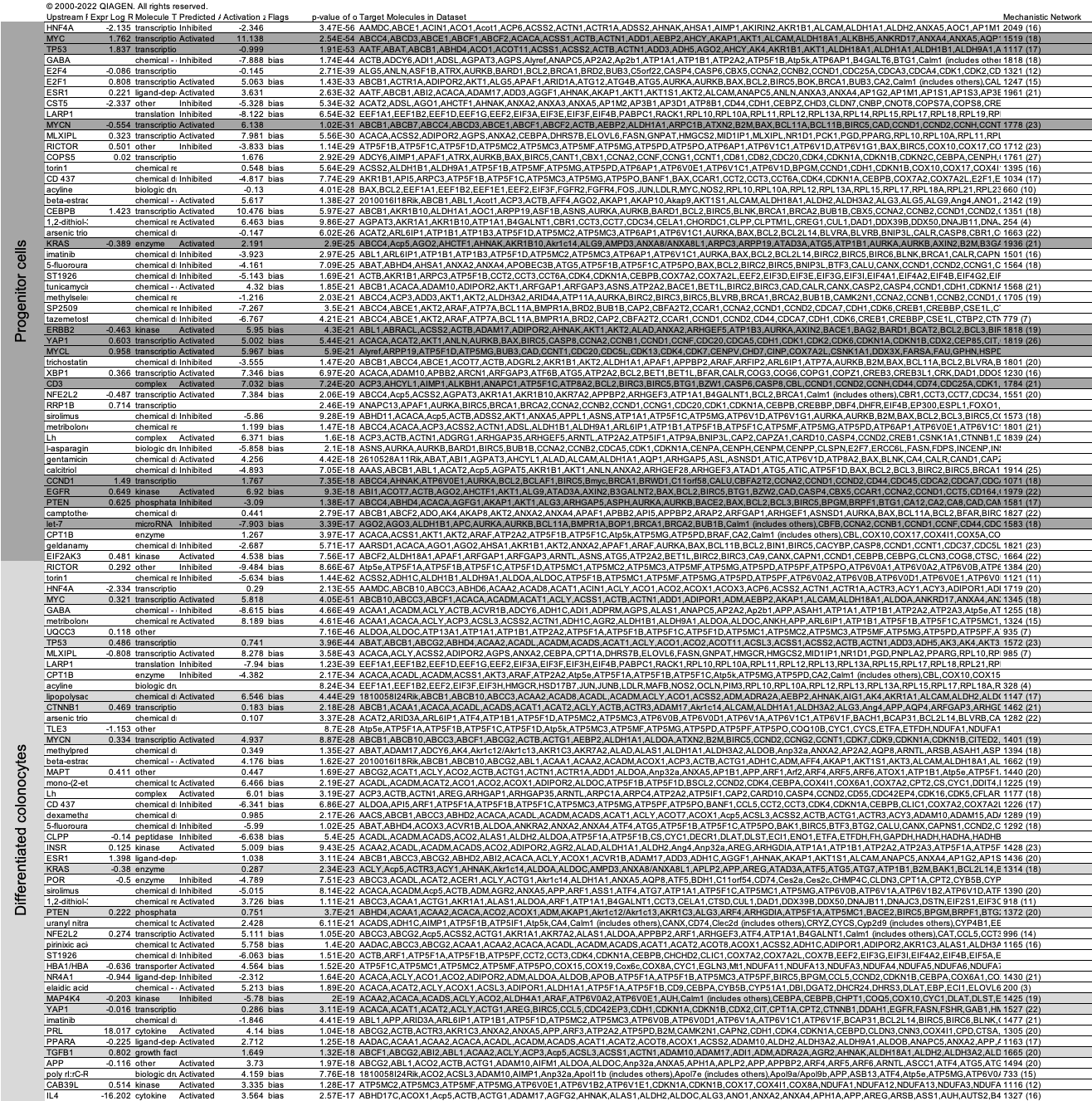


**Table S3. Top 50 hits for Ingenuity Pathway Analysis (IPA) upstream regulator prediction of (top) progenitor and (bottom) differentiated colonocytes.** Shaded rows represent specific upstream regulators of interest that were subsequently plotted for the IPA analysis.

**Table S4. List of antibodies used for high-dimensional flow cytometry.**

1. Perez J, Springthorpe VS, Sattar SA. Clospore: a liquid medium for producing high titers of semi-purified spores of Clostridium difficile. J AOAC Int **2011**;94(2):618-26.

2. Lyras D, O'Connor JR, Howarth PM, Sambol SP, Carter GP, Phumoonna T*, et al.* Toxin B is essential for virulence of Clostridium difficile. Nature **2009**;458(7242):1176-9 doi 10.1038/nature07822.

3. Macedonia MC, Drewes JL, Markham NO, Simmons AJ, Roland JT, Vega PN*, et al.* Clinically adaptable polymer enables simultaneous spatial analysis of colonic tissues and biofilms. NPJ Biofilms Microbiomes **2020**;6(1):33 doi 10.1038/s41522-020-00143-x.

4. Bloedt K, Riecker M, Poppert S, Wellinghausen N. Evaluation of new selective culture media and a rapid fluorescence in situ hybridization assay for identification of Clostridium difficile from stool samples. J Med Microbiol **2009**;58(Pt 7):874-7 doi 10.1099/jmm.0.009811-0.

5. Drewes JL, White JR, Dejea CM, Fathi P, Iyadorai T, Vadivelu J*, et al.* High-resolution bacterial 16S rRNA gene profile meta-analysis and biofilm status reveal common colorectal cancer consortia. NPJ Biofilms Microbiomes **2017**;3:34 doi 10.1038/s41522-017-0040-3.

6. Markham NO, Bloch SC, Shupe JA, Laubacher EN, Thomas AK, Kroh HK*, et al.* Murine Intrarectal Instillation of Purified Recombinant Clostridioides difficile Toxins Enables Mechanistic Studies of Pathogenesis. Infect Immun **2021**;89(4) doi 10.1128/IAI.00543-20.

7. Chung L, Thiele Orberg E, Geis AL, Chan JL, Fu K, DeStefano Shields CE*, et al.* Bacteroides fragilis Toxin Coordinates a Pro-carcinogenic Inflammatory Cascade via Targeting of Colonic Epithelial Cells. Cell Host Microbe **2018**;23(2):203-14 e5 doi 10.1016/j.chom.2018.01.007.

8. Bolger AM, Lohse M, Usadel B. Trimmomatic: a flexible trimmer for Illumina sequence data. Bioinformatics **2014**;30(15):2114-20 doi 10.1093/bioinformatics/btu170.

9. Magoc T, Salzberg SL. FLASH: fast length adjustment of short reads to improve genome assemblies. Bioinformatics **2011**;27(21):2957-63 doi 10.1093/bioinformatics/btr507.

10. Caporaso JG, Kuczynski J, Stombaugh J, Bittinger K, Bushman FD, Costello EK*, et al.* QIIME allows analysis of high-throughput community sequencing data. Nat Methods **2010**;7(5):335-6 doi 10.1038/nmeth.f.303.

11. Edgar RC. Search and clustering orders of magnitude faster than BLAST. Bioinformatics **2010**;26(19):2460-1 doi 10.1093/bioinformatics/btq461.

12. Langmead B, Wilks C, Antonescu V, Charles R. Scaling read aligners to hundreds of threads on general-purpose processors. Bioinformatics **2019**;35(3):421-32 doi 10.1093/bioinformatics/bty648.

13. Wang Q, Garrity GM, Tiedje JM, Cole JR. Naive Bayesian classifier for rapid assignment of rRNA sequences into the new bacterial taxonomy. Appl Environ Microbiol **2007**;73(16):5261-7 doi 10.1128/AEM.00062-07.

14. Daquigan N, Seekatz AM, Greathouse KL, Young VB, White JR. High-resolution profiling of the gut microbiome reveals the extent of Clostridium difficile burden. NPJ Biofilms Microbiomes **2017**;3:35 doi 10.1038/s41522-017-0043-0.

15. Shaikh FY, White JR, Gills JJ, Hakozaki T, Richard C, Routy B*, et al.* A Uniform Computational Approach Improved on Existing Pipelines to Reveal Microbiome Biomarkers of Nonresponse to Immune Checkpoint Inhibitors. Clin Cancer Res **2021**;27(9):2571-83 doi 10.1158/1078-0432.CCR-20-4834.

16. Martinson JN, Broadaway S, Lohman E, Johnson C, Alam MJ, Khaleduzzaman M*, et al.* Evaluation of portability and cost of a fluorescent PCR ribotyping protocol for Clostridium difficile epidemiology. J Clin Microbiol **2015**;53(4):1192-7 doi 10.1128/JCM.03591-14.

17. Snydman DR, McDermott LA, Jenkins SG, Goldstein EJC, Patel R, Forbes BA*, et al.* Epidemiologic trends in Clostridioides difficile isolate ribotypes in United States from 2011 to 2016. Anaerobe **2020**;63:102185 doi 10.1016/j.anaerobe.2020.102185.

18. Tomkovich S, Dejea CM, Winglee K, Drewes JL, Chung L, Housseau F*, et al.* Human colon mucosal biofilms from healthy or colon cancer hosts are carcinogenic. J Clin Invest **2019**;129(4):1699-712 doi 10.1172/JCI124196.

19. Aitken SL, Alam MJ, Khaleduzzaman M, Walk ST, Musick WL, Pham VP*, et al.* In the Endemic Setting, Clostridium difficile Ribotype 027 Is Virulent But Not Hypervirulent. Infect Control Hosp Epidemiol **2015**;36(11):1318-23 doi 10.1017/ice.2015.187.
