# Supplementary material for: Human Colon Cancer–Derived Clostridioides difficile Strains Drive Colonic Tumorigenesis in Mice
Source: Cancer Discov. 2022 Jun 9;12(8):1873–85. doi: 10.1158/2159-8290.CD-21-1273 (PMC9357196; doi:10.1158/2159-8290.CD-21-1273)
Supplement: Supplementary Table [file cd-21-1273_table_s1_suppst1.pdf]

Mouse identifier

Isolates cultured from distal colons (tightly adherent)

Bacterial species	Day 4 M155 D	Day 4 M156 D	Day 7 M160 D	Day 7 M161 D	Wk 2 M166 D	Wk 2 M167 D	Wk 4 M171 D	Wk 4 M172 D	Wk 8 M176 D	Wk 8 M177 D
Bacteroides dorei	■	■	■	■	■	■		■	■	■
Erysipelatoclostridium ramosum[1]	■	■	■	■	■	■	■	■	■	■
Clostridioides difficile[2]	■	■	■	■					■	■
Clostridium_sp_7_2_43F AA (from sequencing)	■	■			■	■	■	■	■	■
Clostridium innocuum	■		■				■	■	■	■
Enterococcus gallinarum						■	■			
Enteroccocus hirae	■	■	■	■				■		
Ruminococcus gnavus			■	■	■		■	■	■	■
Klebsiella pneumoniae	■	■	■			■				
Parabacteroides distasonis							■	■	■	■
Clostridium aldenense	■		■				■			
Enterrococcus faecium		■		■		■				
Blautia coccoides	■				■		■	■		
Enterococcus avium	■	■							■	
Robinsoniella peoriensis				■				■		■
Clostridium butyricum	■			■						
Hungatella hathewayi[3]					■					■
Clostridium cadaveris	■									
Clostridium disporicum	■	■								
Alistipes onderdonkii										
Clostridium symbiosum										
Coprobacillus cateniformis							■			
Flavonifractor plautii		■					■			
Micrococcus luteus						■				
Terrisporobacter glycolicus										
Bacillus cereus[4]		■								
Bacillus circulans										
Gordonibacter pamelae	■									
Bifidobacterium longum[4]							■			
Brevibacillus agri[4]						■				
Clostridium intestinale										
Enterococcus faecalis										
Paraeggerthella hongkongensis (from sequencing)		■								
Staphylococcus epidermidis[4]							■			

Isolates cultured from distal colon washes (loosely adherent)

Bacterial species	Day 4 M155 W	Day 4 M156 W	Day 7 M160 W	Day 7 M161 W	Wk 2 M166 W	Wk 2 M167 W	Wk 4 M171 W	Wk 4 M172 W	Wk 8 M176 W	Wk 8 M177 W
Bacteroides dorei		■	■	■	■	■	■	■	■	■
Erysipelatoclostridium ramosum[1]		■	■		■		■	■		
Clostridioides difficile[2]	■	■	■		■	■	■	■		
Clostridium_sp_7_2_43F AA (from sequencing)	■				■	■	■	■	■	■
Clostridium innocuum					■		■	■	■	■
Enterococcus gallinarum	■		■		■	■	■		■	■
Enteroccocus hirae	■	■	■	■	■			■		■
Ruminococcus gnavus			■	■	■			■		■
Klebsiella pneumoniae	■	■	■	■		■		■		
Parabacteroides distasonis	■						■	■	■	■
Clostridium aldenense					■			■		
Enterrococcus faecium		■	■	■	■	■				
Blautia coccoides										
Enterococcus avium	■	■				■			■	
Robinsoniella peoriensis							■	■		
Clostridium butyricum	■	■		■						
Hungatella hathewayi[3]						■				■
Clostridium cadaveris	■			■						
Clostridium disporicum										
Alistipes onderdonkii								■		■
Clostridium symbiosum		■		■						
Coprobacillus cateniformis							■			■
Flavonifractor plautii										
Micrococcus luteus					■					
Terrisporobacter glycolicus	■				■					
Bacillus cereus[4]										
Bacillus circulans						■				
Gordonibacter pamelae										
Bifidobacterium longum[4]										
Brevibacillus agri[4]										
Clostridium intestinale										■
Enterococcus faecalis				■						
Paraeggerthella hongkongensis (from sequencing)										
Staphylococcus epidermidis[4]										

[1] Formerly Clostridium ramosum
[2] Formerly Clostridium difficile
[3] Formerly Clostridium hathewayi
[4] Not included in 3728T isolate mixture

Table S1
