# Supplementary material for: Human Colon Cancer–Derived Clostridioides difficile Strains Drive Colonic Tumorigenesis in Mice
Source: Cancer Discov. 2022 Jun 9;12(8):1873–85. doi: 10.1158/2159-8290.CD-21-1273 (PMC9357196; doi:10.1158/2159-8290.CD-21-1273)
Supplement: Supplementary Table [file cd-21-1273_table_s2_suppst2.pdf]

C. difficile strain (CIm = Clinical Isolate from germ-free mouse)	TcdA status	TcdB status	Toxin status by Vero cell assay (detects both TcdA and TcdB)	Binary toxin status (cdtA / cdtB)	Ribotype	Source (CRC = colorectal cancer, BF = biofilm, T = tumor, GF = germ-free, CDI = *C. difficile* infection)	Notes on source	Reference
CIm161_DC1_3728T (CIm_3728T)	+	+	+	-/-	485	CRC patient BF+T -> GF mouse	3728T slurry-gavaged GF Min mice (distal colon tissue from mouse 161, distal colon isolate 1)	This study
CIm2663_BF+T mix (CIm_3728T)	+	+	+	-/-	485	CRC patient BF+T mix -> GF mouse	BF+T mixed slurry-gavaged GF Min mice (stool from mouse 2663 at University of Florida)	This study; Tomkovich and Dejea et al. 2019
CIm313_3752T	+	+	+	-/-	014-020	CRC patient BF+T -> GF mouse	3752T slurry-gavaged GF Min mice (stool from mouse 313)	This study
ATCC 700057 (VPI 11186)	-	-	nd	-/-	038		ATCC	
ATCC 9689 (90556-M6S)	+	+	nd	-/-	001		ATCC	
630∆erm WT	+	+	nd	-/-	012	CDI patient	Parent 630 strain was isolated from a patient with pseudomembranous colitis and was associated with a hospital outbreak in Switzerland in 1979-1980; current strain has been extensively lab passaged	Wust et al. 1982; Lyras et al. 2009
630∆erm tcdB-	+	-	nd	-/-	012	CDI patient		Lyras et al. 2009
M7404 WT (BI/NAP1/027)	+	+	+	+/+	027	CDI patient	Canadian epidemic strain	Carter et al. 2015
M7404 tcdA-	-	+	+	+/+	027	CDI patient		Carter et al. 2015
M7404 tcdB-	+	-	+	+/+	027	CDI patient		Carter et al. 2015
M7404 tcdA-B-	-	-	-	+/+	027	CDI patient		Carter et al. 2015

Table S2
