# Supplementary material for: Human Colon Cancer–Derived Clostridioides difficile Strains Drive Colonic Tumorigenesis in Mice
Source: Cancer Discov. 2022 Jun 9;12(8):1873–85. doi: 10.1158/2159-8290.CD-21-1273 (PMC9357196; doi:10.1158/2159-8290.CD-21-1273)
Supplement: Supplementary Table [file cd-21-1273_table_s4_suppst4.pdf]

Staining	Target	Fluorophore	Clone
Viability dye	Aqua	V510	NA
Surface	CD45.2	UV805	104
Surface	THY1.2	UV395	53-2.1
Surface	CD3e	UV496	145-2C11
Surface	gdTCR	AF488	GL3
Surface	CD4	V421	GK1.5
Surface	CD8	APC-Cy7	53-6.7
Surface	CD19	APC-Cy7	1D3
Surface	Ly-6A/E (Sca-1)	BUV563	D7
Surface	IL-33R (ST2)	PE-Cy7	RMST2-33
Surface	IL-25R	efluor660	MUNC33
Surface	Nkp46	AF700/R700	29A1.4
Surface	PD-1	BB700	J43
Surface	CXCR5	UV737	2G8
Surface	CD11c	V650	HL3
Surface	I-E/A	V570	M5/114.15.2
Surface	CD103	UV615	M290
Surface	CD11b	V750	M1/70
Surface	f4/80	V711	T45-2342
Surface	Ly6G	UV395	1A8
Surface	Ly6C	APC-Cy7	AL-21
Intracellular	FOXP3	AF488	FJK16S
Intracellular	TBET	BB790	O4-46
Intracellular	EOMES	PECF594	X4-83
Intracellular	GATA3	BB630	L50-823
Intracellular	RORGT	PE-cy5	Q31-378
Intracellular	BCL-6	BB660	K112-91
Intracellular	IFNg	BV605	xmg1.2
Intracellular	IL-17A	V786	TC11-18H10
Intracellular	IL-4	UV661	11B11
Intracellular	IL-22	PE	1H8PWSR

Table S4
